# Association Between the Dietary Inflammatory Index and Cardiorenal Anemia Syndrome: Evidence From NHANES and Related‐Phenotype Exploration in KNHANES

**DOI:** 10.1002/fsn3.72232

**Published:** 2026-09-02

**Authors:** Shirong Lin, Yufeng Chen, Yadan Chen, Lihan Wu, Weijia Zeng, Haiyang Song, Wenjun Ding, Xiaoxiangxin Luo, Jun Ke, Feng Chen

**Affiliations:** ^1^ Shengli Clinical Medical College of Fujian Medical University Fuzhou Fujian China; ^2^ Department of Emergency Fujian Provincial Hospital Fuzhou Fujian China; ^3^ Fuzhou University Affiliated Provincial Hospital Fuzhou Fujian China; ^4^ Fujian Provincial Key Laboratory of Emergency Medicine Fuzhou Fujian China; ^5^ The First Affiliated Hospital of Xiamen University Xiamen Fujian China; ^6^ Xiamen University Xiamen Fujian China

**Keywords:** cardiorenal anemia syndrome, Dietary Inflammatory Index, inflammation indices, machine learning, SHAP

## Abstract

Cardiorenal anemia syndrome (CRAS) links heart failure, chronic kidney disease, and anemia, but the role of diet‐related inflammation remains unclear. In the National Health and Nutrition Examination Survey (NHANES), we examined associations of the Dietary Inflammatory Index (DII) with CRAS, dose–response patterns, inflammatory mediation, and machine‐learning prediction, alongside analysis of a myocardial infarction–CKD–anemia phenotype in the Korea National Health and Nutrition Examination Survey (KNHANES). This study included 25,388 NHANES and 32,935 KNHANES adults. Survey‐weighted regression and restricted cubic splines assessed associations with CRAS in NHANES and the related phenotype in KNHANES. Exploratory mediation evaluated six inflammatory indices. In NHANES participants with heart failure, least absolute shrinkage and selection operator regression selected predictors for 12 internally validated models, with cross‐phenotype application in KNHANES and interpretation using Shapley additive explanations (SHAP). Higher DII was associated with greater odds of CRAS in NHANES (odds ratio [OR] = 1.22, 95% CI: 1.07–1.40) and of the related phenotype in KNHANES (OR = 1.19, 95% CI: 1.10–1.29). The association was largely linear in NHANES but nonlinear in KNHANES. In NHANES, all six indices showed positive indirect associations, with estimated proportions ranging from 5.22% to 16.26%. CatBoost showed the highest discrimination in NHANES internal validation (ROC‐AUC = 0.741; PR‐AUC = 0.556) and in the KNHANES cross‐phenotype application (ROC‐AUC = 0.753; PR‐AUC = 0.698). Higher DII was associated with greater odds of CRAS in NHANES, with directionally consistent findings for the related phenotype in KNHANES. The model may support model‐based CRAS identification and showed preliminary applicability to the related phenotype.

## Graphical abstract

Higher Dietary Inflammatory Index scores were associated with greater odds of cardiorenal anemia syndrome in NHANES and of a related phenotype in KNHANES. Exploratory mediation analyses showed positive indirect associations through six systemic inflammatory indices. Among 12 machine‐learning algorithms, CatBoost showed the strongest discrimination for identifying cardiorenal anemia syndrome among NHANES participants with heart failure and also performed best in the exploratory application to the related phenotype in KNHANES. These findings connect diet‐related inflammation with cardiorenal anemia syndrome and support further evaluation of model‐based identification across populations.

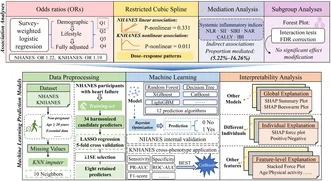

## 1 Introduction

Cardiorenal anemia syndrome (CRAS) is defined as a vicious cycle characterized by a complex interplay among heart failure (HF), chronic kidney disease (CKD), and anemia. The term was first formally introduced by Silverberg et al. in the early 21st century to describe this complex clinical entity (Silverberg et al. 2000, 2004). Globally, CRAS represents a significant public health challenge. Epidemiological data indicate that among patients with chronic heart failure, the prevalence of CRAS ranges from 19% to 44% (Rangaswami et al. 2019). Its widespread nature is corroborated by reports from various regions; for instance, a study in Kenya reported a high prevalence of anemia and renal dysfunction comorbidity among patients with heart failure (Mwololo 2013), while another study in the Middle East found that nearly one‐third of heart failure patients met the diagnostic criteria for CRAS (Manla et al. 2023). Despite the well‐documented clinical consequences of CRAS, its underlying drivers and mediating mechanisms remain an area of intense research interest and ongoing challenge.

Inflammation contributes to the development and progression of HF, CKD, and anemia (Mann 2015; Kadatane et al. 2023). Diet may influence this process by modulating systemic inflammatory activity (Kiecolt‐Glaser et al. 2012; Naderinejad et al. 2020). The Dietary Inflammatory Index (DII) was developed to quantify the overall inflammatory potential of an individual's diet and is widely used in nutritional epidemiology (Hébert et al. 2019). Unlike assessments of individual foods or nutrients, it summarizes the combined pro‐ and anti‐inflammatory contributions of the diet.

Given the role of inflammation in cardiac, renal, and hematologic disorders, a higher DII may be associated with CRAS, and systemic inflammation may partly account for this association. However, population‐based evidence on the association between DII and CRAS remains limited (Palazzuoli et al. 2011; Shivappa et al. 2017). Single biomarkers such as C‐reactive protein capture only one aspect of the inflammatory response (Zhang and Ni 2011). Composite indices derived from routine blood measurements may provide a broader measure of immune and inflammatory status and have been associated with cardiovascular, renal, and other chronic diseases (Tuzimek et al. 2024; Islam et al. 2024; Ma et al. 2023). These include the neutrophil‐to‐lymphocyte ratio (NLR), systemic immune‐inflammation index (SII), systemic inflammation response index (SIRI), neutrophil‐to‐albumin ratio (NAR), C‐reactive protein–albumin–lymphocyte index (CALLY), and inflammatory burden index (IBI).

Beyond identifying epidemiologic associations, recognizing CRAS among individuals with HF remains clinically challenging. CRAS involves complex and potentially nonlinear relationships among dietary, lifestyle, and clinical factors that may not be fully captured by conventional regression models (McCullough 2021). Machine‐learning methods can model such relationships and may improve prediction, although their incremental value over clinical benchmarks requires evaluation (Shehab et al. 2022; Rahmani et al. 2021). KNHANES also provides an opportunity to examine a related MI–CKD–anemia phenotype in a population with different dietary and clinical characteristics.

Using NHANES, we examined the association between DII and HF‐defined CRAS, including dose–response patterns, subgroup analyses, and exploratory mediation by six systemic inflammatory indices. We then developed and compared machine‐learning models for CRAS among participants with HF. KNHANES was used to examine the corresponding DII association and to explore model performance for a related MI–CKD–anemia phenotype.

## 2 Methods

### 2.1 Study Population

This study was a secondary cross‐sectional analysis of publicly available data from two repeated, nationally representative health examination surveys: the National Health and Nutrition Examination Survey (NHANES) and the Korea National Health and Nutrition Examination Survey (KNHANES). NHANES is an ongoing, nationally representative survey program conducted by the National Center for Health Statistics (NCHS), part of the Centers for Disease Control and Prevention (CDC) (Ahluwalia et al. 2016). Our primary analysis utilized data from 10 consecutive NHANES survey cycles spanning from 1999 to 2018, which initially included 132,518 participants. KNHANES, a nationally representative survey administered by the Korea Disease Control and Prevention Agency (KDCA) (Kweon et al. 2014), provided a complementary national setting for the exploratory analysis of the MI–CKD–anemia phenotype across different dietary and sociocultural contexts. Specifically, KNHANES data collected between 2013 and 2024 were used for the exploratory analysis, including 89,968 participants before exclusions.

**FIGURE 1:**
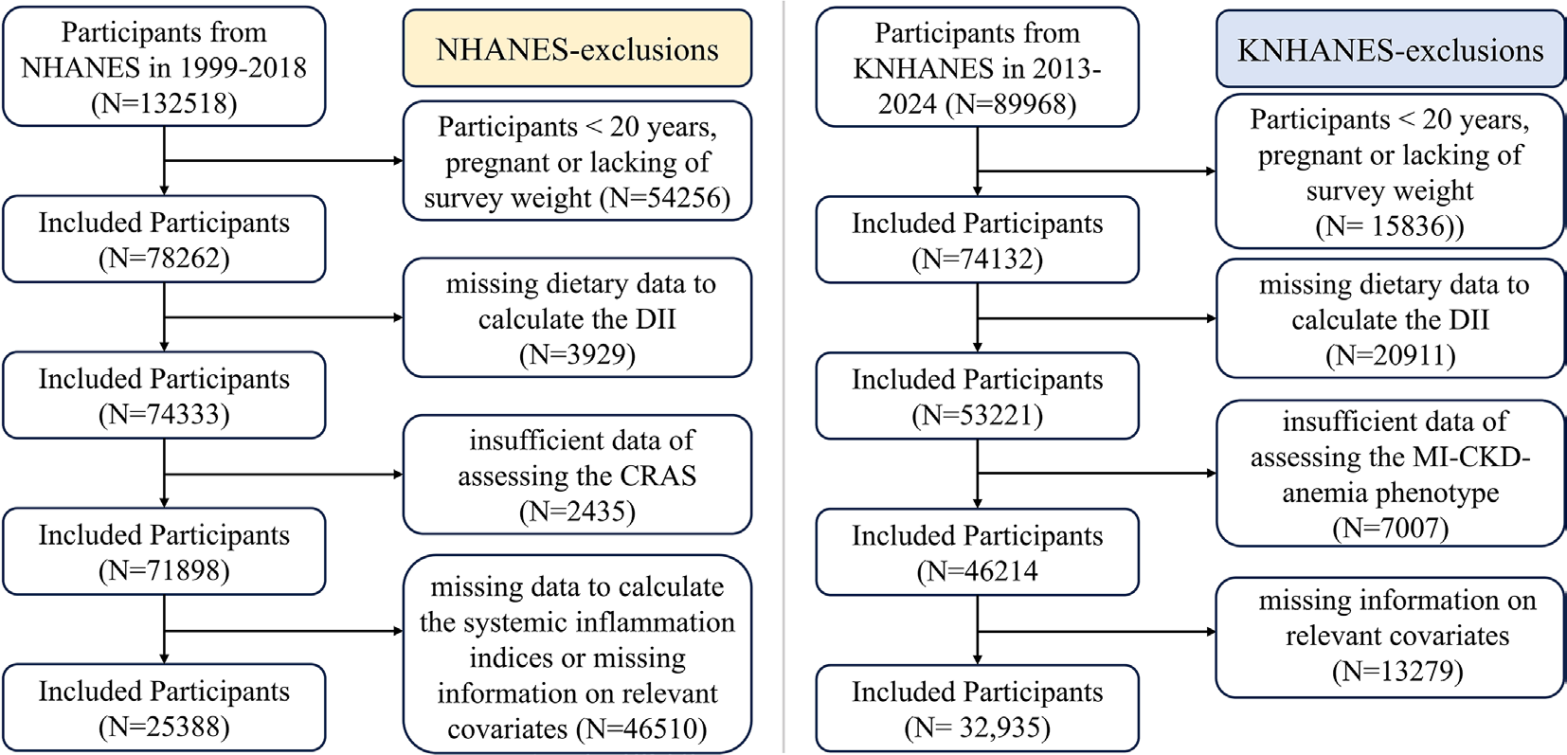
Flowchart of participant inclusion and exclusion process.

The participant selection processes for NHANES (1999–2018) and KNHANES (2013–2024) are summarized in Figure 1. Briefly, participants were excluded if they were under 20 years of age, were pregnant, had insufficient dietary data for DII calculation, lacked information required to define CRAS in NHANES or the MI–CKD–anemia phenotype in KNHANES, or had missing data required to calculate systemic inflammatory indices or relevant covariates. After applying these criteria, 25,388 participants were included in the final NHANES analytical sample, and 32,935 participants were included in the KNHANES analytical sample.

### 2.2 Definitions of HF, CKD, Anemia, and CRAS

In NHANES, heart failure (HF) was identified from a questionnaire‐based self‐reported physician diagnosis. Participants who answered “yes” to the item asking whether a doctor or other health professional had ever told them that they had congestive heart failure (MCQ160B) were classified as having HF (National Center for Health Statistics 2020).

CKD was operationally defined using Kidney Disease: Improving Global Outcomes (KDIGO)‐aligned thresholds as an estimated glomerular filtration rate (eGFR) < 60 mL/min/1.73 m^2^ or a urinary albumin‐to‐creatinine ratio (UACR) ≥ 30 mg/g (Stevens et al. 2024). eGFR was calculated using the 2009 Chronic Kidney Disease Epidemiology Collaboration (CKD‐EPI) creatinine equation (Levey et al. 2009). eGFR categories were defined as G1 (≥ 90), G2 (60–89), G3a (45–59), G3b (30–44), G4 (15–29), and G5 (< 15 mL/min/1.73 m^2^). Albuminuria categories were defined as A1 (< 30 mg/g), A2 (30–300 mg/g), and A3 (> 300 mg/g) (Stevens et al. 2024).

Anemia was defined according to the 2011 World Health Organization (WHO) hemoglobin thresholds as a hemoglobin level of < 13.0 g/dL in men and < 12.0 g/dL in women (World Health Organization 2011).

In NHANES, CRAS was operationalized as the coexistence of HF, CKD, and anemia. Participants meeting all three criteria were classified as having CRAS; all others were classified as non‐CRAS. In machine‐learning analyses restricted to NHANES participants with HF, CRAS was defined as the coexistence of CKD and anemia. For the complementary KNHANES analysis, a related cardio–renal–hematologic outcome was operationalized as the coexistence of physician‐diagnosed myocardial infarction (DI5_dg), CKD, and anemia and was termed the MI–CKD–anemia phenotype. The CKD and anemia definitions were identical to those used in NHANES.

### 2.3 Calculation of the Dietary Inflammation Index

The Dietary Inflammatory Index (DII) is a comprehensive, literature‐based scoring tool developed to quantify the overall pro‐inflammatory or anti‐inflammatory potential of an individual's diet. The index was first proposed by Shivappa et al. (2014). The calculation of the DII followed the standardized procedure established by its developers. Briefly, the intake of each dietary parameter was standardized against a global reference database and converted into a centered percentile score. This score was then multiplied by the corresponding inflammatory effect score, which was derived from a comprehensive literature review. Finally, the scores for all dietary parameters were summed to yield an overall DII score for each participant (Shivappa et al. 2015). To characterize the directional contributions to the overall score, DII_pro was calculated as the sum of the pro‐inflammatory parameter scores, whereas DII_anti_abs was calculated as the absolute value of the sum of the anti‐inflammatory parameter scores. Previous construct‐validation studies have also reported associations between DII scores calculated from reduced sets of available dietary parameters and inflammatory biomarkers in the SEASONS, Asklepios, and HELENA cohorts (Hébert et al. 2019; Shivappa et al. 2017, 2015). A higher DII score indicates a more potent pro‐inflammatory diet, whereas a lower or negative score suggests a stronger anti‐inflammatory potential. For categorical analysis, participants were classified into two groups based on their DII scores: a pro‐inflammatory diet group (DII > 0) and an anti‐inflammatory diet group (DII ≤ 0) (Wirth et al. 2016).

Dietary intake data for DII calculation were obtained from 24‐h dietary recalls in both surveys. In NHANES, one in‐person recall was available for the 1999–2002 cycles, whereas two recalls, comprising an initial in‐person interview and a subsequent telephone interview, were available from 2003 onward. Mean intake across the two recall days was used when both were available (Shu et al. 2022). In KNHANES, dietary intake was based on a single 24‐h recall conducted as part of the nutrition survey (Kweon et al. 2014). In NHANES, the DII score was computed based on 27 available dietary parameters: alcohol, vitamin B12, vitamin B6, β‐carotene, caffeine, carbohydrates, cholesterol, total energy, total fat, dietary fiber, folic acid, iron, magnesium, monounsaturated fatty acids (MUFAs), niacin, protein, polyunsaturated fatty acids (PUFAs), riboflavin, saturated fat, selenium, thiamin, vitamin A, vitamin C, vitamin E, zinc, n‐3 fatty acids, and n‐6 fatty acids. For the exploratory KNHANES analysis (2013–2024), the DII score was calculated using the same standardized procedure but was based on 22 available dietary parameters due to the unavailability of five components (alcohol, vitamin B12, vitamin B6, selenium, and caffeine). The specific inflammatory effect scores for all dietary parameters used in this study are provided in Table S1. To assess the influence of parameter availability on cross‐cohort comparability, we additionally recalculated DII in NHANES using the same 22 dietary parameters available in KNHANES.

Data on complete blood counts and blood biochemistry parameters were obtained from the Mobile Examination Center (MEC). Six systemic inflammation indices—NLR, SII, SIRI, NAR, CALLY, and IBI—were calculated using the following formulas (Liu et al. 2025; Wu et al. 2025):

- NLR = Neutrophil count/Lymphocyte count
- SII = Platelet count × Neutrophil count/Lymphocyte count
- SIRI = Neutrophil count × Monocyte count/Lymphocyte count
- NAR = Neutrophil count/Serum albumin concentration
- CALLY = Serum albumin concentration × Lymphocyte count/C‐reactive protein concentration
- IBI = C‐reactive protein concentration × Neutrophil count/Lymphocyte count

### 2.4 Covariates and Feature Extraction

The covariates included in this study were categorized into demographic characteristics, lifestyle factors, and comorbidities. Demographic variables included age, sex, ethnicity, marital status, poverty‐income ratio (PIR), body mass index (BMI), and education level. Lifestyle habits included smoking status, alcohol consumption, and physical activity. Comorbidities of interest were hypertension, diabetes mellitus (DM), and hyperlipidemia. Detailed definitions for each of these variables are available in Table S2.

For machine‐learning model development, candidate predictors were harmonized across datasets to support the exploratory cross‐phenotype application. The feature pool comprised nutrient‐intake variables together with key demographic, lifestyle, and comorbidity covariates that were consistently available and could be comparably coded in both NHANES and KNHANES. Predictors that were unavailable or could not be harmonized across the two surveys were excluded before model development, leaving 34 candidate variables for feature selection and model training. The eight predictors retained during feature selection were directly available in both datasets under the harmonized definitions.

### 2.5 Statistical Analysis and Model Development

Survey‐weighted analyses accounted for the complex, multistage sampling designs of NHANES and KNHANES (Hu et al. 2025). Survey strata and primary sampling units were specified using SDMVSTRA and SDMVPSU for NHANES and kstrata and psu for KNHANES, with primary sampling units nested within strata. For the 10 NHANES cycles pooled from 1999 to 2018, the 1999–2002 4‐year MEC weight was divided by 5, and the 2‐year MEC weights for the cycles from 2003–2004 through 2017–2018 were divided by 10. For KNHANES 2013–2024, the integrated survey weight (w_tot), covering the health interview, health examination, and nutrition survey components, was used as provided without additional rescaling. Survey weights were applied to the descriptive and association analyses but not to the machine‐learning analyses.

In NHANES, baseline characteristics were compared between participants with and without CRAS. In KNHANES, baseline characteristics were compared between participants with and without the MI–CKD–anemia phenotype. For continuous variables, weighted *t*‐tests were used, and data are presented as weighted means ± standard deviations (SDs). For categorical variables, weighted chi‐square tests were employed, with data presented as counts and weighted percentages (*n*, %). Before imputation, variable‐specific missingness and joint missingness patterns were summarized separately for each cohort. Covariates with missing values were imputed using the K‐nearest neighbors (KNN) method (Altamimi et al. 2024). Detailed missing‐data summaries are provided in Table S3. The six systemic inflammation indices were natural log‐transformed (ln‐transformed) to address their skewed distributions (Ma et al. 2024). The skewness and data distribution of these indices before and after transformation are shown in Figure S1.

Survey‐weighted logistic regression models were used to estimate the association of DII with CRAS in NHANES and with the MI–CKD–anemia phenotype in KNHANES. The associations of the six systemic inflammatory indices with CRAS were examined in NHANES. Odds ratios (ORs) and 95% confidence intervals (CIs) were reported (Liu et al. 2023). Three hierarchical models were fitted with sequential covariate adjustment: Model I was unadjusted, Model II was adjusted for demographic variables, and Model III was adjusted for all covariates. Restricted cubic spline (RCS) models with three knots were fitted for each cohort‐specific outcome to explore potential nonlinearity (Gauthier et al. 2020). Subgroup analyses were conducted separately in each cohort to assess potential effect modification of the DII association with the corresponding outcome. Exploratory mediation analyses were conducted to evaluate whether systemic inflammatory indices statistically explained part of the association between DII and CRAS. These analyses estimated the total effect (TE), direct effect (DE), and indirect effect (IE). Confidence intervals for the mediation estimates were obtained from 1000 bootstrap resamples (Huang et al. 2024).

Furthermore, three sensitivity analyses were performed in NHANES to evaluate the robustness of the main findings: (1) multiple imputation was used as an alternative method for handling missing data; (2) participants with extreme BMI values were excluded; and (3) complete blood count and blood biochemistry parameters were additionally included as covariates in the fully adjusted model: albumin (g/dL), C‐reactive protein (CRP, mg/dL), lymphocyte (10^9^/L), monocyte (10^9^/L), neutrophils (10^9^/L), and platelet (×10^9^/L).

To specifically assess the influence of the different numbers of available DII parameters, the same survey‐weighted logistic regression models and restricted cubic spline analysis were repeated in NHANES using the 22‐component score based on the parameters shared with KNHANES. Pearson correlation was used to compare the continuous 27‐ and 22‐component scores, and quadratic weighted kappa was used to assess agreement in quartile classification. The 95% confidence interval for kappa was estimated using 2000 bootstrap resamples.

The robustness of the indirect association through SII was further assessed across sequential covariate‐adjustment models and after excluding participants with Ln_SII values below the 1st or above the 99th percentile. Multicollinearity was evaluated using generalized variance inflation factors (GVIFs), expressed as GVIF^(1/(2 × df)), where df denotes degrees of freedom. Detailed model specifications are provided in Table S4.

To account for multiple testing in the exploratory analyses, the Benjamini–Hochberg false discovery rate (FDR) procedure was applied within related families of tests, including DII subscore analyses, inflammatory‐index associations, indirect effects, spline tests, and subgroup interaction tests within each cohort. Nominal and FDR‐adjusted *p* values are reported in Table S5, with an FDR‐adjusted *p* value below 0.05 considered statistically significant.

To develop the CRAS prediction model, 746 NHANES participants with heart failure were randomly divided using stratified sampling into a development training set (70%, *n* = 522) and an internal validation set (30%, *n* = 224). All preprocessing and feature selection procedures were fitted exclusively within the development training set. The 34 harmonized candidate predictors were standardized using parameters estimated from the training set, and feature selection was performed using least absolute shrinkage and selection operator (LASSO) regression. Five‐fold cross‐validation and the one‐standard‐error rule were used to determine the regularization parameter, thereby favoring a more parsimonious predictor set (Guan et al. 2024). The standardization parameters estimated from the NHANES development training set and the selected feature set were then fixed and applied unchanged to the NHANES internal validation set and KNHANES.

Following feature selection, 12 machine‐learning algorithms were developed and compared: logistic regression, random forest, extreme gradient boosting (XGBoost), light gradient‐boosting machine (LightGBM), gradient boosting decision tree (GBDT), decision tree, KNN, support vector machine (SVM), adaptive boosting (AdaBoost), categorical boosting (CatBoost), multilayer perceptron (MLP), and Gaussian naive Bayes. Hyperparameters were optimized within the NHANES development training set using BayesSearchCV, with ROC‐AUC as the optimization metric. Each Bayesian search comprised 20 iterations, with one candidate configuration evaluated per iteration. Nested stratified five‐fold cross‐validation was used: the outer folds generated out‐of‐fold predictions, and the inner folds selected hyperparameters. Each algorithm subsequently underwent a final stratified five‐fold Bayesian search using the complete development training set. A random seed of 42 was used for data splitting and stochastic procedures, with deterministic fold‐specific seeds derived from this value. The complete hyperparameter search spaces and final selected settings are provided in Table S6. No synthetic oversampling or other class‐balancing resampling procedure was applied, and the observed outcome distributions were retained in the training and evaluation datasets. For each model, a classification threshold was determined from the NHANES training‐set out‐of‐fold predictions by maximizing the Youden index and was subsequently applied unchanged to the NHANES internal validation set and KNHANES.

After fitting the models in the NHANES development training set, predictive performance was evaluated in the held‐out NHANES internal validation set. Models were ranked by ROC‐AUC in the held‐out NHANES internal validation set, and the highest‐ranking algorithm was selected for subsequent interpretation and clinical benchmark comparison. The fitted models were subsequently applied to the MI–CKD–anemia phenotype in KNHANES as an exploratory cross‐phenotype assessment. No model recalibration, refitting, or cohort‐specific threshold adjustment was performed in KNHANES. The training‐derived standardization parameters, fitted NHANES models, and model‐specific classification thresholds were applied unchanged. Model discrimination was evaluated using ROC‐AUC with 95% confidence intervals derived from 2000 participant‐level bootstrap resamples and area under the precision–recall curve (PR‐AUC), summarized using average precision. The Brier score was calculated to assess overall probabilistic accuracy. Accuracy, sensitivity, specificity, and F1‐score were calculated using the model‐specific thresholds fixed from the NHANES training set out‐of‐fold predictions. Precision–recall curves, calibration curves, decision curve analysis (DCA), and clinical impact curves (CIC) were also generated for the NHANES internal validation and exploratory KNHANES cross‐phenotype settings (Kerr et al. 2016).

To assess incremental performance, the selected CatBoost model was compared with an age‐only logistic regression model and a conventional clinical logistic regression model including age, BMI, sex, smoking status, hypertension, diabetes mellitus, and hyperlipidemia. The benchmark models were developed in the NHANES training set and evaluated in the same NHANES and KNHANES settings as CatBoost, using thresholds derived from five‐fold out‐of‐fold predictions. Paired differences in ROC‐AUC, PR‐AUC, and Brier score were estimated using 2000 participant‐level bootstrap resamples.

Finally, Shapley Additive Explanations (SHAP) were calculated for the selected CatBoost model in the NHANES internal validation set (Nohara et al. 2022). A combined SHAP beeswarm and mean absolute SHAP summary plot was used to characterize global feature contributions. Representative true‐positive and true‐negative participants were selected for individual SHAP force plots. To assess the stability of global feature‐importance rankings, we performed 1000 participant‐level bootstrap resamples of the NHANES internal validation set. For each feature, we calculated the percentile 95% confidence interval of the mean absolute SHAP value and the median bootstrap rank. As a descriptive measure of stability among the leading predictors, we also calculated the proportion of bootstrap samples in which each feature remained among the three highest‐ranked features.

Survey‐based statistical analyses were performed in R version 4.1.1, and machine‐learning analyses were conducted in Python version 3.11.5 using scikit‐learn version 1.4.2 and the corresponding algorithm‐specific packages. A two‐sided *p* value < 0.05 was considered statistically significant.

## 3 Results

### 3.1 Baseline Characteristics of Participants

Before imputation, variable‐specific missingness ranged from 0% to 6.94% in NHANES and from 0% to 4.01% in KNHANES. Detailed variable‐specific and joint missingness patterns are provided in Table S3. A total of 25,388 participants were included in this study, among whom 239 were identified as having CRAS. The baseline characteristics of the study population stratified by DII quartiles are summarized in Table 1. Participants in the highest DII quartile exhibited a higher prevalence of CRAS, were more likely to be female, had higher educational attainment, and were more likely to be current smokers. The distributions of the six systemic inflammatory indices also varied across DII quartiles.

**TABLE 1:** Baseline characteristics of participants stratified by Dietary Inflammatory Index quartiles in NHANES.

Characteristic	Overall	Q1	Q2	Q3	Q4	*p*
	*N* = 25,388	*N* = 6347	*N* = 6347	*N* = 6347	*N* = 6347	
DII	0.89 ± (1.74)	−1.35 ± (0.90)	0.51 ± (0.39)	1.75 ± (0.34)	3.08 ± (0.52)	< 0.001
Age, years	46.17 ± (16.12)	47.24 ± (15.69)	46.44 ± (15.86)	45.72 ± (16.16)	45.10 ± (16.80)	< 0.001
PIR	3.20 ± (1.61)	3.57 ± (1.54)	3.30 ± (1.59)	3.11 ± (1.58)	2.73 ± (1.62)	< 0.001
Physical activity, (MET/week)	3242.13 ± (5402.82)	3276.48 ± (5225.53)	3278.32 ± (5364.48)	3093.85 ± (5170.12)	3318.32 ± (5872.71)	< 0.001
BMI, kg/m^2^	28.50 ± (6.41)	27.65 ± (5.98)	28.51 ± (6.23)	28.88 ± (6.59)	29.09 ± (6.80)	< 0.001
IBI	7.51 ± (23.96)	5.39 ± (13.25)	7.46 ± (29.62)	8.04 ± (24.09)	9.51 ± (26.18)	< 0.001
CALLY	103.13 ± (166.01)	120.89 ± (165.34)	102.83 ± (151.89)	93.60 ± (180.16)	92.63 ± (164.71)	< 0.001
SIRI	1.23 ± (0.82)	1.20 ± (0.76)	1.23 ± (0.82)	1.23 ± (0.81)	1.27 ± (0.89)	0.020
NAR	0.10 ± (0.04)	0.09 ± (0.03)	0.10 ± (0.04)	0.10 ± (0.04)	0.11 ± (0.04)	< 0.001
SII	551.49 ± (320.65)	520.10 ± (286.48)	546.88 ± (321.09)	558.09 ± (318.49)	586.81 ± (355.07)	< 0.001
NLR	2.17 ± (1.08)	2.14 ± (1.02)	2.17 ± (1.10)	2.16 ± (1.03)	2.22 ± (1.17)	0.120
Sex						
Male	13,350 (51.2%)	4179 (64.9%)	3647 (56.1%)	3092 (45.5%)	2432 (35.6%)	< 0.001
Female	12,038 (48.8%)	2168 (35.1%)	2700 (43.9%)	3255 (54.5%)	3915 (64.4%)	
Ethnicity						
Non‐Hispanic White	12,832 (74.0%)	3438 (77.2%)	3293 (75.1%)	3094 (72.4%)	3007 (70.5%)	< 0.001
Non‐Hispanic Black	4750 (9.0%)	838 (5.7%)	1032 (7.6%)	1378 (10.9%)	1502 (12.5%)	
Mexican American	3915 (6.7%)	991 (6.7%)	1018 (6.9%)	953 (6.6%)	953 (6.6%)	
Other Hispanic	1823 (4.7%)	426 (4.3%)	456 (4.5%)	455 (4.8%)	486 (5.2%)	
Other race	2068 (5.7%)	654 (6.1%)	548 (6.0%)	467 (5.2%)	399 (5.3%)	
Marital status						
Married/Living with partner	15,865 (66.3%)	4289 (70.5%)	4127 (68.7%)	3886 (65.3%)	3563 (59.6%)	< 0.001
Widowed/Divorced/Separated	4996 (16.5%)	999 (13.3%)	1179 (15.4%)	1273 (16.7%)	1545 (21.3%)	
Never married	4527 (17.3%)	1059 (16.3%)	1041 (16.0%)	1188 (18.0%)	1239 (19.1%)	
Education						
Below high school	2010 (3.6%)	338 (2.4%)	480 (3.3%)	536 (4.1%)	656 (4.8%)	< 0.001
High school	8905 (32.0%)	1712 (23.1%)	2096 (29.5%)	2298 (33.5%)	2799 (43.5%)	
Above high school	14,473 (64.4%)	4297 (74.4%)	3771 (67.2%)	3513 (62.4%)	2892 (51.7%)	
Smoking status						
Never	13,642 (54.2%)	3611 (57.8%)	3418 (54.2%)	3436 (55.0%)	3177 (49.2%)	< 0.001
Former	6567 (25.6%)	1886 (29.9%)	1750 (27.4%)	1549 (23.6%)	1382 (20.5%)	
Now	5179 (20.2%)	850 (12.3%)	1179 (18.4%)	1362 (21.4%)	1788 (30.3%)	
Drinking alcohol						
Never	3007 (9.6%)	638 (8.0%)	687 (8.9%)	743 (9.8%)	939 (12.1%)	< 0.001
Former	4025 (13.0%)	832 (10.9%)	929 (12.0%)	1035 (12.7%)	1229 (16.9%)	
Mild	9089 (38.0%)	2732 (45.8%)	2337 (38.6%)	2182 (36.4%)	1838 (29.7%)	
Moderate	4142 (18.2%)	959 (16.9%)	1086 (18.8%)	1051 (18.4%)	1046 (19.0%)	
Heavy	5125 (21.1%)	1186 (18.4%)	1308 (21.5%)	1336 (22.6%)	1295 (22.3%)	
Hypertension						
Yes	10,043 (34.4%)	2362 (33.0%)	2482 (34.5%)	2551 (35.5%)	2648 (34.8%)	0.119
No	15,345 (65.6%)	3985 (67.0%)	3865 (65.5%)	3796 (64.5%)	3699 (65.2%)	
DM						
No	19,877 (82.8%)	5075 (83.8%)	4987 (82.1%)	4929 (82.6%)	4886 (82.6%)	0.01
Yes	3714 (10.7%)	822 (10.0%)	889 (10.3%)	969 (11.0%)	1034 (11.4%)	
Prediabetes	1797 (6.6%)	450 (6.1%)	471 (7.6%)	449 (6.4%)	427 (6.1%)	
Hyperlipidemia						
Yes	18,081 (70.3%)	4313 (67.4%)	4511 (69.5%)	4563 (71.7%)	4694 (73.3%)	< 0.001
No	7307 (29.7%)	2034 (32.6%)	1836 (30.5%)	1784 (28.3%)	1653 (26.7%)	
CRAS						
Yes	239 (0.5%)	28 (0.2%)	43 (0.4%)	71 (0.6%)	97 (0.9%)	< 0.001
No	25,149 (99.5%)	6319 (99.8%)	6304 (99.6%)	6276 (99.4%)	6250 (99.1%)	

For the complementary KNHANES analysis, 32,935 participants were included, among whom 556 met the criteria for the MI–CKD–anemia phenotype. The baseline characteristics across DII quartiles are summarized in Table S7. In KNHANES, the prevalence of the related phenotype increased across DII quartiles (from 0.7% in Q1 to 1.8% in Q4; *p* < 0.001) and higher DII quartiles were generally associated with lower PIR and lower physical activity.

For the development of the CRAS prediction model among patients with heart failure, baseline characteristics were further compared between those with and without CRAS. Compared with non‐CRAS participants, those with CRAS were older, had higher prevalences of hypertension, hyperlipidemia, and diabetes, and reported lower dietary intake of magnesium and zinc. Additionally, levels of five systemic inflammation indices were higher in the CRAS group. More detailed information is provided in Table S8.

In the exploratory KNHANES related‐phenotype analysis, participants with the MI–CKD–anemia phenotype were older and had lower PIR and physical activity levels, and higher DII values than participants without this phenotype. They also had higher prevalences of hypertension and diabetes and generally lower intakes of multiple nutrients, as summarized in Table S9.

### 3.2 Associations of DII With CRAS and the MI–CKD–Anemia Phenotype

**FIGURE 2:**
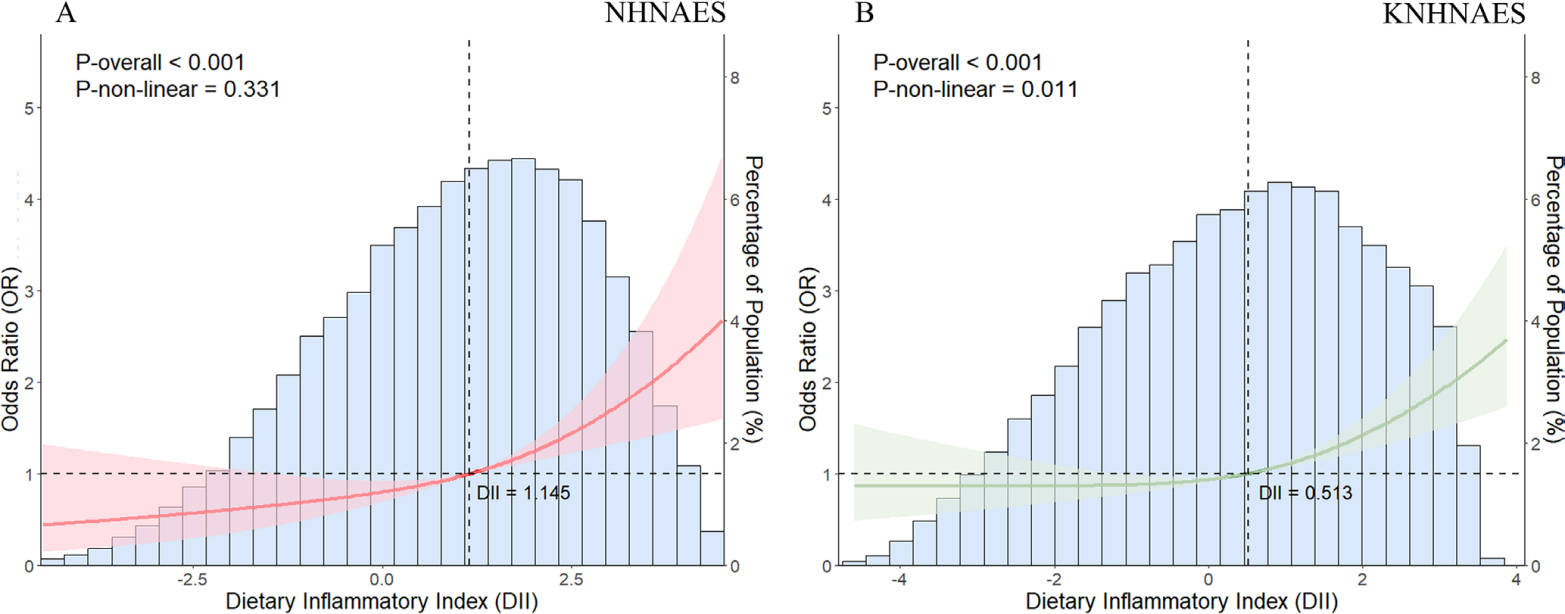
Restricted cubic spline analyses of DII in relation to CRAS in NHANES and the MI–CKD–anemia phenotype in KNHANES. (A) NHANES cohort. (B) KNHANES cohort.

As shown in Table 2, weighted logistic regression analyses indicated that higher DII was associated with greater odds of CRAS in NHANES and with greater odds of the related phenotype in KNHANES. In the fully adjusted model (Model III), each 1‐point increase in DII was associated with greater odds of CRAS in NHANES (OR = 1.22, 95% CI: 1.07–1.40; *p* = 0.004) and of the related phenotype in KNHANES (OR = 1.19, 95% CI: 1.10–1.29; *p* < 0.001). When analyzed by DII quartiles, participants in the highest quartile had higher odds of CRAS in NHANES and of the related phenotype in KNHANES, with significant dose–response trends. Directionally consistent associations were also observed for DII_pro and DII_anti_abs. Based on this, we constructed restricted cubic spline (RCS) models to characterize the association of DII with the corresponding outcome in each cohort. As shown in Figure 2, after adjustment for all covariates, DII was positively associated with CRAS in NHANES and with the related phenotype in KNHANES (both *p*‐overall < 0.001). In the NHANES cohort, no significant departure from linearity was observed (*p*‐nonlinear > 0.05). In contrast, KNHANES showed evidence of a nonlinear association (*p*‐nonlinear = 0.011), with the odds of the MI–CKD–anemia phenotype increasing more markedly at higher DII values. The FDR‐adjusted results supported the overall spline associations and KNHANES nonlinearity, while the association in NHANES remained largely linear; results for the secondary DII components were also unchanged, as shown in Table S5.

**TABLE 2:** Associations of the Dietary Inflammatory Index (DII) with CRAS in NHANES and the MI–CKD–anemia phenotype in KNHANES using survey‐weighted logistic regression models.

	Model 1		Model 2		Model 3	
	OR (95% CI)	*p*	OR (95% CI)	*p*	OR (95% CI)	*p*
NHANES						
DII	1.38 (1.24, 1.54)	< 0.001	1.30 (1.14, 1.47)	< 0.001	1.22 (1.07, 1.40)	0.004
Q1 (ref)	1		1		1	
Q2	1.44 (0.73, 2.81)	0.29	1.33 (0.68, 2.58)	0.403	1.15 (0.58, 2.30)	0.684
Q3	3.38 (2.10, 5.43)	< 0.001	2.97 (1.81, 4.86)	< 0.001	2.42 (1.47, 3.99)	< 0.001
Q4	4.31 (2.67, 6.96)	< 0.001	3.21 (1.94, 5.29)	< 0.001	2.44 (1.46, 4.09)	0.001
DII_anti_abs	0.53 (0.35, 0.81)	< 0.001	0.61 (0.41, 0.91)	0.015	0.71 (0.48, 1.04)	0.051
DII_pro	1.48 (1.31, 1.68)	< 0.001	1.37 (1.18, 1.60)	< 0.001	1.28 (1.08, 1.52)	0.005
*p* for trend		< 0.001		< 0.001		< 0.001
KNHANES						
DII	1.27 (1.18, 1.35)	< 0.001	1.20 (1.12, 1.30)	< 0.001	1.19 (1.10, 1.29)	< 0.001
Q1 (ref)	1		1		1	
Q2	1.37 (0.99, 1.88)	0.056	1.25 (0.89, 1.74)	0.196	1.19 (0.85, 1.68)	0.317
Q3	1.70 (1.25, 2.31)	< 0.001	1.39 (1.00, 1.93)	0.047	1.28 (0.92, 1.80)	0.147
Q4	2.67 (1.97, 3.61)	< 0.001	2.10 (1.49, 2.94)	< 0.001	2.01 (1.42, 2.84)	< 0.001
DII_anti_abs	0.71 (0.61, 0.83)	< 0.001	0.80 (0.68, 0.92)	0.003	0.82 (0.70, 0.95)	0.008
DII_pro	1.42 (1.30, 1.55)	< 0.001	1.33 (1.20, 1.47)	< 0.001	1.32 (1.19, 1.46)	< 0.001
*p* for trend		< 0.001		< 0.001		< 0.001

### 3.3 Associations Between the Six Systemic Inflammation Indices and CRAS

The results of weighted logistic regression analyses examining the associations between the six systemic inflammation indices and CRAS in NHANES are presented in Table S10. After adjustment for all covariates, Ln_SIRI, Ln_NLR, Ln_SII, Ln_NAR, and Ln_IBI were significantly and positively associated with CRAS, whereas Ln_CALLY showed a significant negative association. The trend tests were statistically significant (*p* for trend < 0.05). Specifically, each one‐unit increase in Ln_SIRI was associated with a 324% greater odds of CRAS (OR = 4.24, 95% CI: 3.34–5.39, *p* < 0.001); each one‐unit increase in Ln_NLR was associated with a 244% higher odds (OR = 3.44, 95% CI: 2.27–5.23, *p* < 0.001); each one‐unit increase in Ln_SII was associated with 71% higher odds of CRAS (OR = 1.71, 95% CI: 1.08–2.69, *p* = 0.021); each one‐unit increase in Ln_NAR was associated with a 288% higher odds (OR = 3.88, 95% CI: 2.38–6.33, *p* < 0.001); each one‐unit increase in Ln_CALLY was associated with a 54% lower odds (OR = 0.46, 95% CI: 0.39–0.55, *p* < 0.001); and each one‐unit increase in Ln_IBI was associated with a 94% higher odds (OR = 1.94, 95% CI: 1.65–2.27, *p* < 0.001).

**FIGURE 3:**
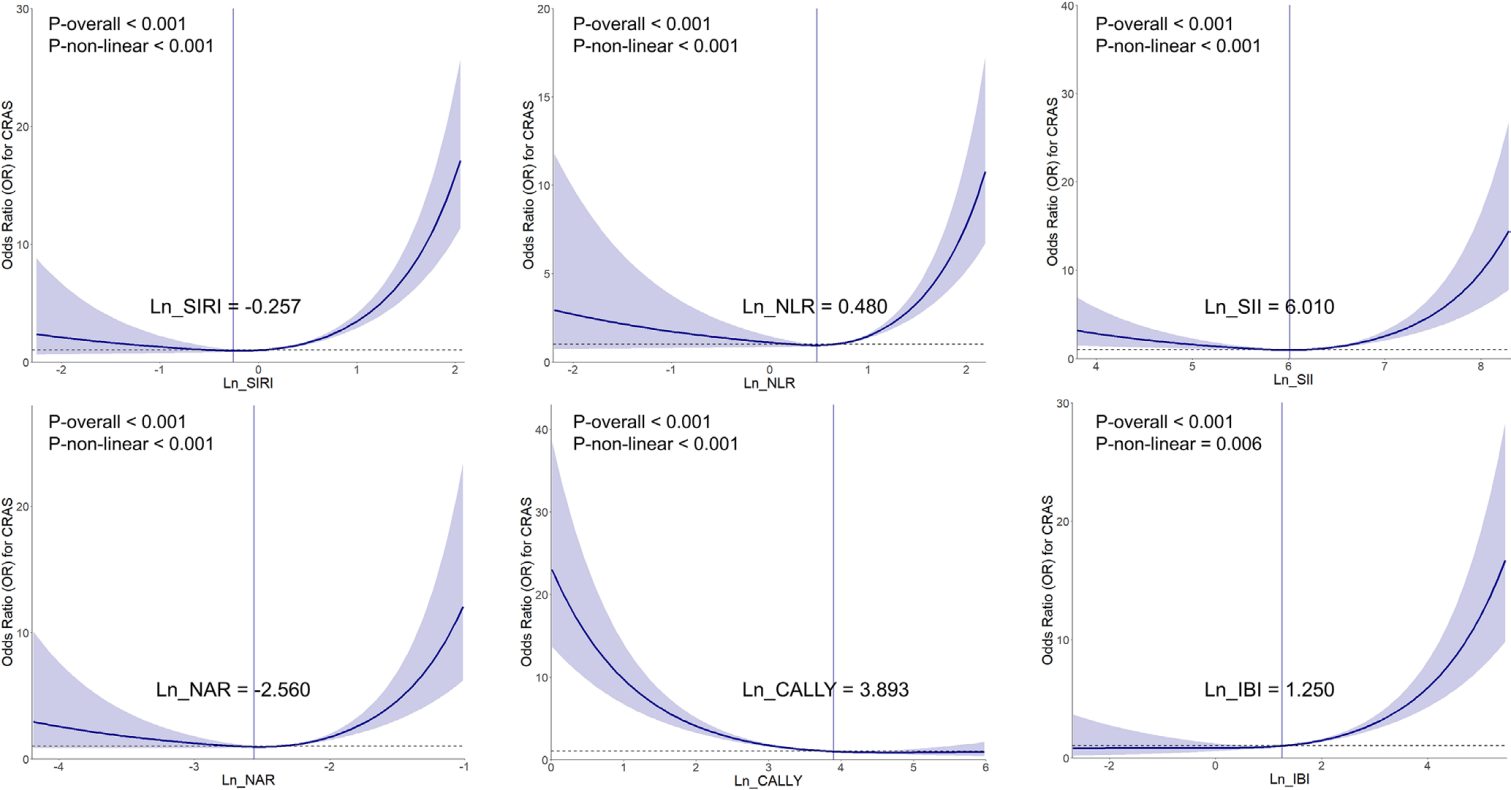
Restricted cubic spline analyses of the associations between six systemic inflammation indices and cardiorenal anemia syndrome.

As shown in Figure 3, restricted cubic spline (RCS) analyses demonstrated nonlinear associations between the six systemic inflammation indices and CRAS (*p*‐overall < 0.001, *p*‐nonlinear < 0.05). The analyses indicated that Ln_SIRI, Ln_NLR, Ln_SII, and Ln_NAR showed approximately U‐shaped associations with the odds of CRAS, whereas Ln_CALLY and Ln_IBI showed inverse and positive associations with the odds of CRAS, respectively.

### 3.4 Exploratory Mediation Analysis of Inflammatory Indices

**FIGURE 4:**
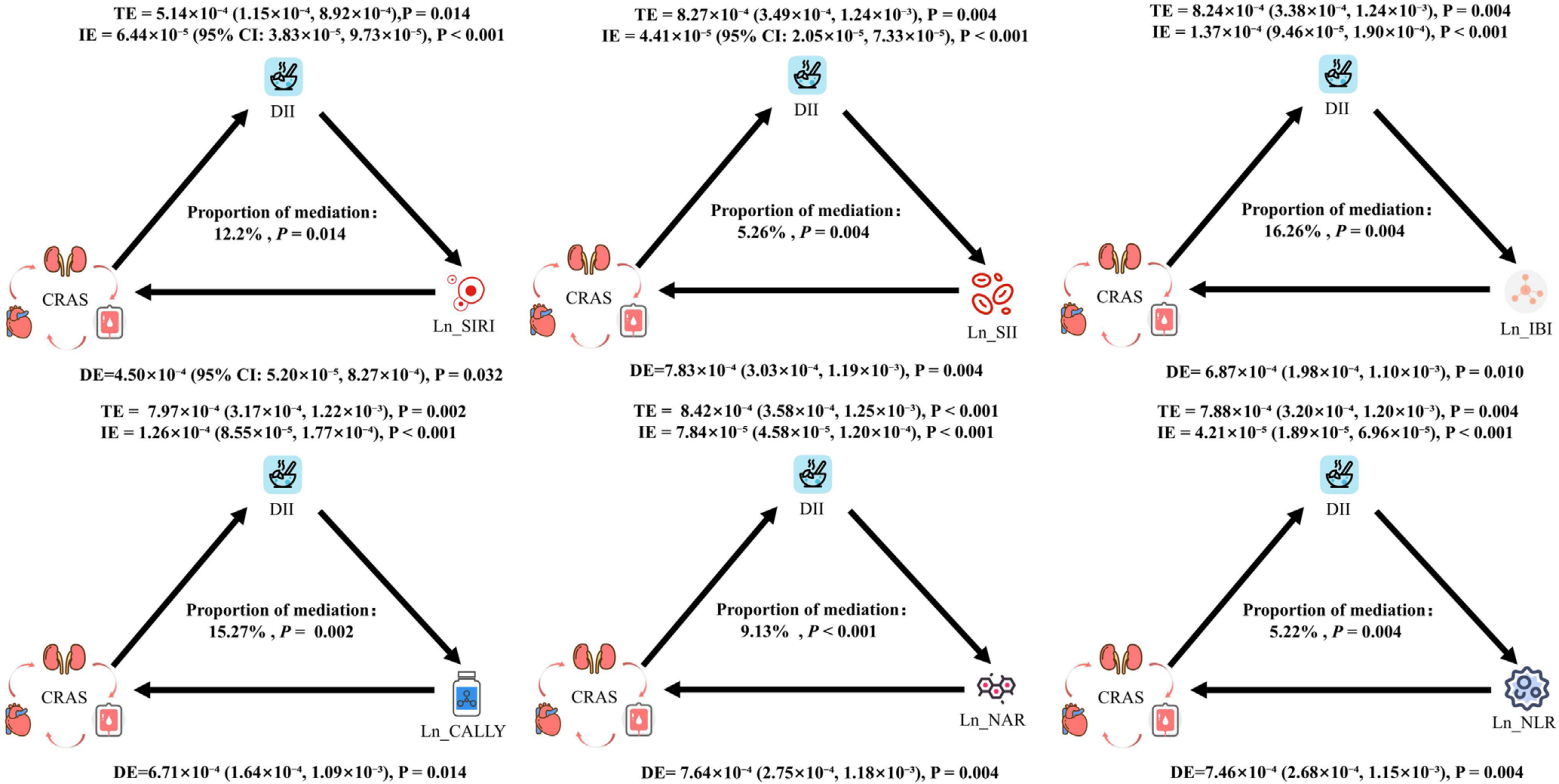
Mediation effects of six systemic inflammation indices in the association between DII and CRAS.

To explore whether systemic inflammatory indices statistically accounted for part of the DII–CRAS association, we conducted exploratory mediation analyses in the NHANES cohort with adjustment for all covariates. As shown in Figure 4, all six indices showed significant positive indirect associations. IBI and CALLY had the largest estimated proportions, accounting for 16.26% (95% CI: 9.11%–42.63%) and 15.27% (95% CI: 8.69%–44.76%), respectively. The corresponding proportions were 12.21% (95% CI: 5.70%–48.12%) for SIRI and 9.13% (95% CI: 4.74%–24.02%) for NAR. SII and NLR showed smaller positive indirect associations, with estimated proportions of 5.26% (95% CI: 2.17%–13.95%; *p* = 0.004) and 5.22% (95% CI: 2.03%–14.50%), respectively. The estimated indirect effects, direct effects, and proportions were statistically significant for all six indices (*p* < 0.05). Detailed mediation results are provided in Table 3. The continuous associations of all six indices, the corresponding spline tests, and all six indirect effects remained statistically significant after FDR correction, as shown in Table S5.

**TABLE 3:** Exploratory mediation results for six systemic inflammatory indices.

Mediator	ACME (95% CI)	ADE (95% CI)	TE (95% CI)	PM (95% CI)
NLR	4.213 × 10^−5^ (1.890 × 10^−5^, 6.965 × 10^−5^)***	7.458 × 10^−4^ (2.682 × 10^−4^, 1.151 × 10^−3^)**	7.879 × 10^−4^ (3.198 × 10^−4^, 1.197 × 10^−3^)**	5.217% (2.032%, 14.498%)**
SII	4.41 × 10^−5^ (2.05 × 10^−5^, 7.33 × 10^−5^)***	7.83 × 10^−4^ (3.03 × 10^−4^, 1.19 × 10^−3^)**	8.27 × 10^−4^ (3.49 × 10^−4^, 1.24 × 10^−3^)**	5.26% (2.17%, 13.95%)**
SIRI	6.437 × 10^−5^ (3.832 × 10^−5^, 9.728 × 10^−5^)***	4.496 × 10^−4^ (5.195 × 10^−5^, 8.266 × 10^−4^)*	5.140 × 10^−4^ (1.147 × 10^−4^, 8.925 × 10^−4^)*	12.214% (5.698%, 48.123%)*
NAR	7.837 × 10^−5^ (4.578 × 10^−5^, 1.197 × 10^−4^)***	7.641 × 10^−4^ (2.752 × 10^−4^, 1.175 × 10^−3^)**	8.424 × 10^−4^ (3.582 × 10^−4^, 1.255 × 10^−3^)***	9.130% (4.741%, 24.023%)***
CALLY	1.262 × 10^−4^ (8.553 × 10^−5^, 1.767 × 10^−4^)***	6.705 × 10^−4^ (1.637 × 10^−4^, 1.088 × 10^−3^)*	7.967 × 10^−4^ (3.170 × 10^−4^, 1.220 × 10^−3^)**	15.274% (8.693%, 44.759%)**
IBI	1.372 × 10^−4^ (9.459 × 10^−5^, 1.897 × 10^−4^)***	6.867 × 10^−4^ (1.984 × 10^−4^, 1.104 × 10^−3^)**	8.239 × 10^−4^ (3.378 × 10^−4^, 1.240 × 10^−3^)**	16.256% (9.113%, 42.628%)**

*Note:* Models were adjusted for age, sex, ethnicity, marital status, PIR, education, smoking status, alcohol use, physical activity, BMI, hypertension, diabetes mellitus, and hyperlipidemia. Confidence intervals were estimated using 1000 bootstrap resamples. These quantities are reported as statistical mediation estimates from a cross‐sectional analysis and are not interpreted as causal effects.

Abbreviations: ACME, average causal mediation effect; ADE, average direct effect; CI, confidence interval.

*p < 0.05, **p < 0.01, ***p < 0.001.

### 3.5 Subgroup Analyses

**FIGURE 5:**
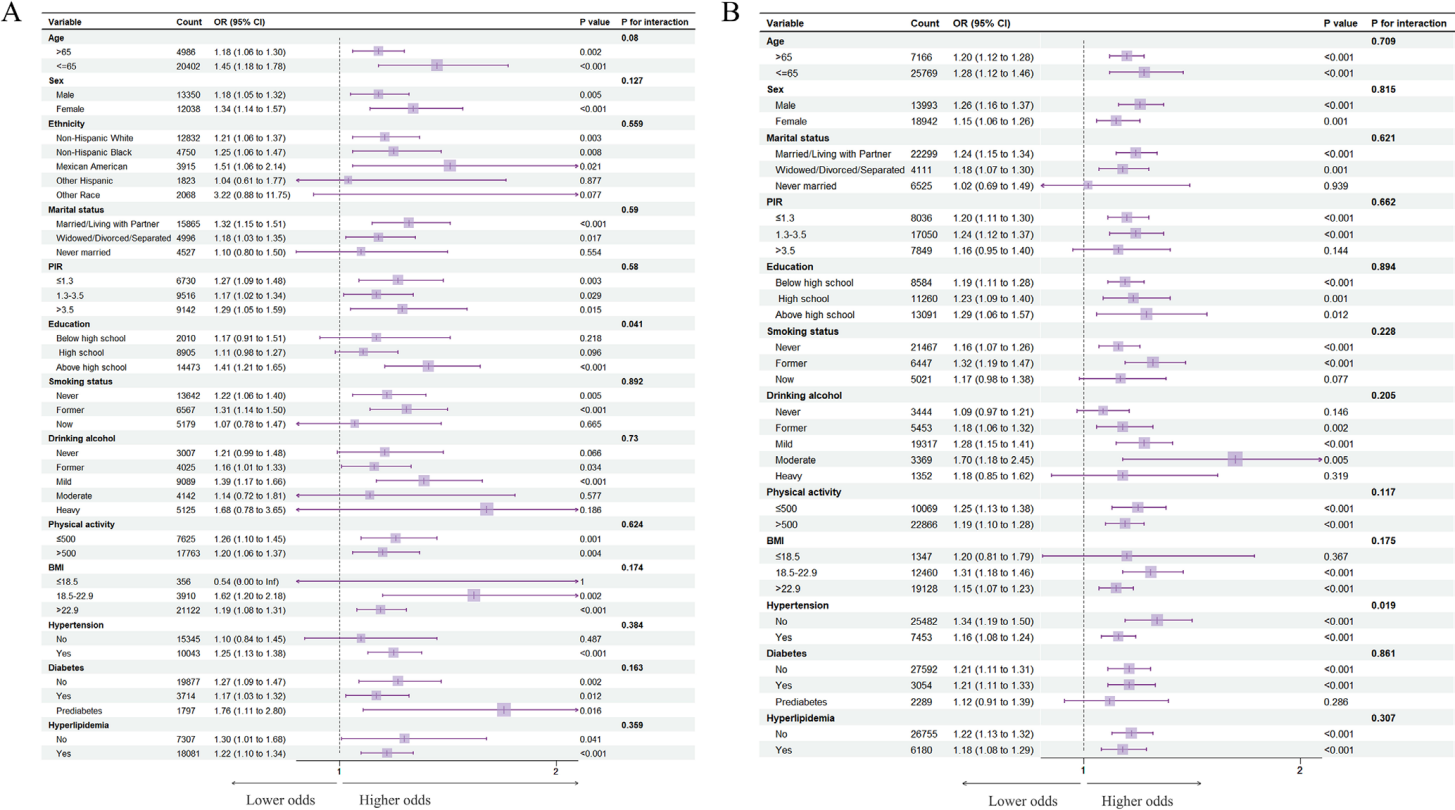
Subgroup analyses of DII with CRAS in NHANES and with the MI–CKD–anemia phenotype in KNHANES. Models were adjusted for all covariates. Interaction *p* values shown in the figure are nominal; corresponding FDR‐adjusted *p* values are reported in Table S5. (A) NHANES cohort. (B) KNHANES cohort.

We conducted subgroup analyses of the DII–CRAS association in NHANES and the DII–MI–CKD–anemia phenotype association in KNHANES, as shown in Figure 5. Overall, the positive associations were broadly consistent across demographic, lifestyle, and clinical strata in both cohorts. In NHANES, the association appeared stronger among participants with education above high school, and the interaction met the nominal significance threshold (*p* for interaction = 0.041), but it was no longer significant after FDR correction (FDR‐adjusted *p* = 0.452). In KNHANES, the association appeared stronger among participants without hypertension (*p* for interaction = 0.019), but this interaction also did not remain significant after correction (FDR‐adjusted *p* = 0.228). No other subgroup interactions were statistically significant. Detailed FDR‐adjusted results are provided in Table S5.

### 3.6 Sensitivity Analyses

We performed sensitivity analyses in the NHANES cohort to assess the robustness of our findings. Specifically, we conducted multiple imputation, excluded participants with extreme BMI values, and additionally adjusted for covariates including complete blood count and biochemical indicators. The positive association between DII and CRAS remained stable across the three sensitivity analyses presented in Table S11. When DII was recalculated in NHANES using the 22 parameters shared with KNHANES, the 22‐component score was strongly correlated with the 27‐component score (Pearson *r* = 0.956; *p* < 0.001) and showed high agreement in quartile classification (quadratic weighted kappa = 0.913, 95% CI: 0.911–0.916). The fully adjusted association remained close to the primary estimate (OR = 1.21, 95% CI: 1.06–1.39; *p* = 0.005; Table S12), and the corresponding restricted cubic spline analysis showed a positive, largely linear association (*p*‐overall < 0.001; *p*‐nonlinear = 0.581; Figure S2).

As shown in Table S4, the positive indirect association through SII also remained consistent across sequential adjustment models, with estimated proportions ranging from 5.19% to 6.08%. After participants with Ln_SII values below the 1st or above the 99th percentile were excluded, the estimated proportion remained similar at 4.63% (95% CI: 1.46%–17.19%; *p* = 0.008). The maximum adjusted GVIF was 1.41 in the mediator model and 1.25 in the outcome model, indicating no substantial multicollinearity.

### 3.7 The Development and Interpretation of Machine Learning Prediction Models

**FIGURE 6:**
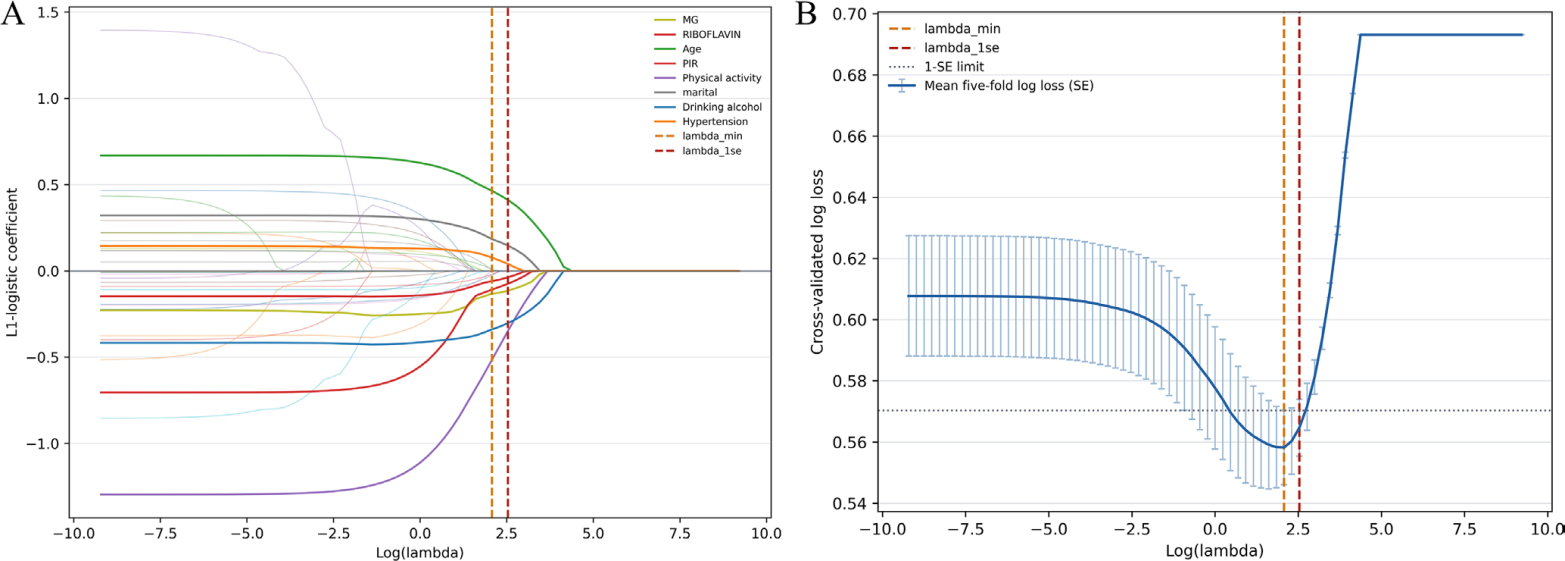
Feature selection using LASSO regression in the NHANES development training set. (A) Coefficient paths across log(*λ*). (B) Five‐fold cross‐validated binomial log loss, with vertical reference lines indicating *λ*min and *λ*1SE.

Feature selection was performed in the NHANES development training set using LASSO regression. The coefficient paths are shown in Figure 6A, and the five‐fold cross‐validation results are presented in Figure 6B. Using the one‐standard‐error rule, eight predictors were retained: magnesium intake (MG), riboflavin intake (RIBOFLAVIN), age, poverty‐income ratio (PIR), physical activity, marital status, drinking alcohol, and hypertension.

Among the 746 NHANES participants with heart failure included in the machine‐learning analysis, 239 had CRAS, corresponding to a prevalence of 32.0%. The prevalence was similar in the development training set (167/522, 32.0%) and the internal validation set (72/224, 32.1%). Performance metrics are summarized in Table 4. In the NHANES internal validation set, CatBoost showed the highest ROC‐AUC (0.7410, 95% CI: 0.6728–0.8049), followed by XGBoost (0.7378, 95% CI: 0.6716–0.8028) and Random Forest (0.7323, 95% CI: 0.6667–0.7979). CatBoost had a PR‐AUC of 0.5564 and the lowest Brier score of 0.1856, whereas XGBoost showed the highest PR‐AUC (0.5867). At its training‐derived threshold, CatBoost achieved an accuracy of 0.6563, sensitivity of 0.6250, specificity of 0.6711, and F1‐score of 0.5389. In the exploratory KNHANES cross‐phenotype application, 556 of 1304 participants (42.6%) had the MI–CKD–anemia phenotype. CatBoost also showed the highest ROC‐AUC (0.7526, 95% CI: 0.7264–0.7773), the highest PR‐AUC (0.6977), and the lowest Brier score (0.2000). Its accuracy, sensitivity, specificity, and F1‐score in KNHANES were 0.6679, 0.7446, 0.6110, and 0.6566, respectively.

**TABLE 4:** Performance of 12 machine‐learning models in the NHANES internal validation set and their exploratory cross‐phenotype application to the MI–CKD–anemia phenotype in KNHANES.

Model	ROC‐AUC (95% CI)	PR‐AUC	Brier score	Accuracy	Sensitivity	Specificity	F1 score
NHANES							
Random Forest	0.732 (0.667–0.798)	0.539	0.196	0.670	0.653	0.678	0.560
XGBoost	0.738 (0.672–0.803)	0.587	0.187	0.679	0.569	0.730	0.532
LightGBM	0.730 (0.664–0.796)	0.575	0.188	0.670	0.458	0.770	0.471
Decision Tree	0.692 (0.621–0.762)	0.549	0.228	0.563	0.778	0.461	0.533
KNN	0.700 (0.629–0.771)	0.487	0.199	0.616	0.736	0.559	0.552
GBDT	0.719 (0.651–0.785)	0.548	0.191	0.652	0.681	0.638	0.557
**CatBoost**	**0.741 (0.673–0.805)**	**0.556**	**0.186**	**0.656**	**0.625**	**0.671**	**0.539**
Logistic Regression	0.731 (0.663–0.795)	0.510	0.205	0.661	0.722	0.632	0.578
SVM	0.718 (0.652–0.785)	0.500	0.192	0.643	0.750	0.592	0.574
AdaBoost	0.719 (0.647–0.786)	0.507	0.234	0.527	0.944	0.329	0.562
MLP	0.725 (0.660–0.791)	0.510	0.194	0.661	0.583	0.697	0.525
Gaussian NB	0.720 (0.652–0.787)	0.494	0.257	0.696	0.528	0.776	0.528
KNHANES							
Random Forest	0.740 (0.713–0.765)	0.667	0.209	0.648	0.781	0.549	0.654
XGBoost	0.745 (0.718–0.770)	0.672	0.203	0.669	0.716	0.634	0.648
LightGBM	0.745 (0.719–0.770)	0.672	0.202	0.676	0.687	0.667	0.644
Decision Tree	0.685 (0.655–0.714)	0.588	0.262	0.615	0.754	0.512	0.625
KNN	0.699 (0.671–0.727)	0.611	0.221	0.623	0.781	0.507	0.639
GBDT	0.748 (0.723–0.773)	0.692	0.201	0.644	0.806	0.524	0.659
**CatBoost**	**0.753 (0.726–0.777)**	**0.698**	**0.200**	**0.668**	**0.745**	**0.611**	**0.657**
Logistic Regression	0.738 (0.711–0.764)	0.686	0.211	0.646	0.768	0.555	0.649
SVM	0.736 (0.710–0.762)	0.686	0.207	0.62	0.811	0.477	0.645
AdaBoost	0.747 (0.720–0.772)	0.683	0.241	0.523	0.978	0.184	0.636
MLP	0.724 (0.697–0.750)	0.671	0.214	0.680	0.685	0.676	0.646
Gaussian NB	0.722 (0.695–0.749)	0.673	0.256	0.666	0.522	0.774	0.571

*Note:* The NHANES HF subgroup contained 239 CRAS cases among 746 participants (32.0%; training, 167/522; internal validation, 72/224), and the KNHANES MI subgroup contained 556 MI–CKD–anemia cases among 1304 participants (42.6%). ROC‐AUC 95% CIs were estimated using 2000 bootstrap resamples, and PR‐AUC was summarized using average precision. Classification metrics used training‐set out‐of‐fold Youden thresholds applied unchanged to both evaluation settings; lower Brier scores indicate better probabilistic accuracy. Bold values indicate the model selected on the basis of overall performance for subsequent interpretation and clinical benchmark comparison.

CatBoost outperformed the age‐only model in terms of ROC‐AUC, PR‐AUC, and Brier score in both evaluation settings, as shown in Table S13. Its performance was also numerically better than that of the conventional clinical model, although the differences were estimated less precisely in the NHANES internal validation set. In KNHANES, CatBoost showed modest improvements in ROC‐AUC and Brier score over the conventional clinical model, whereas the PR‐AUC difference remained uncertain. Paired performance differences are presented in Table S14, and the corresponding ROC and precision–recall curves are shown in Figure S4.

**FIGURE 7:**
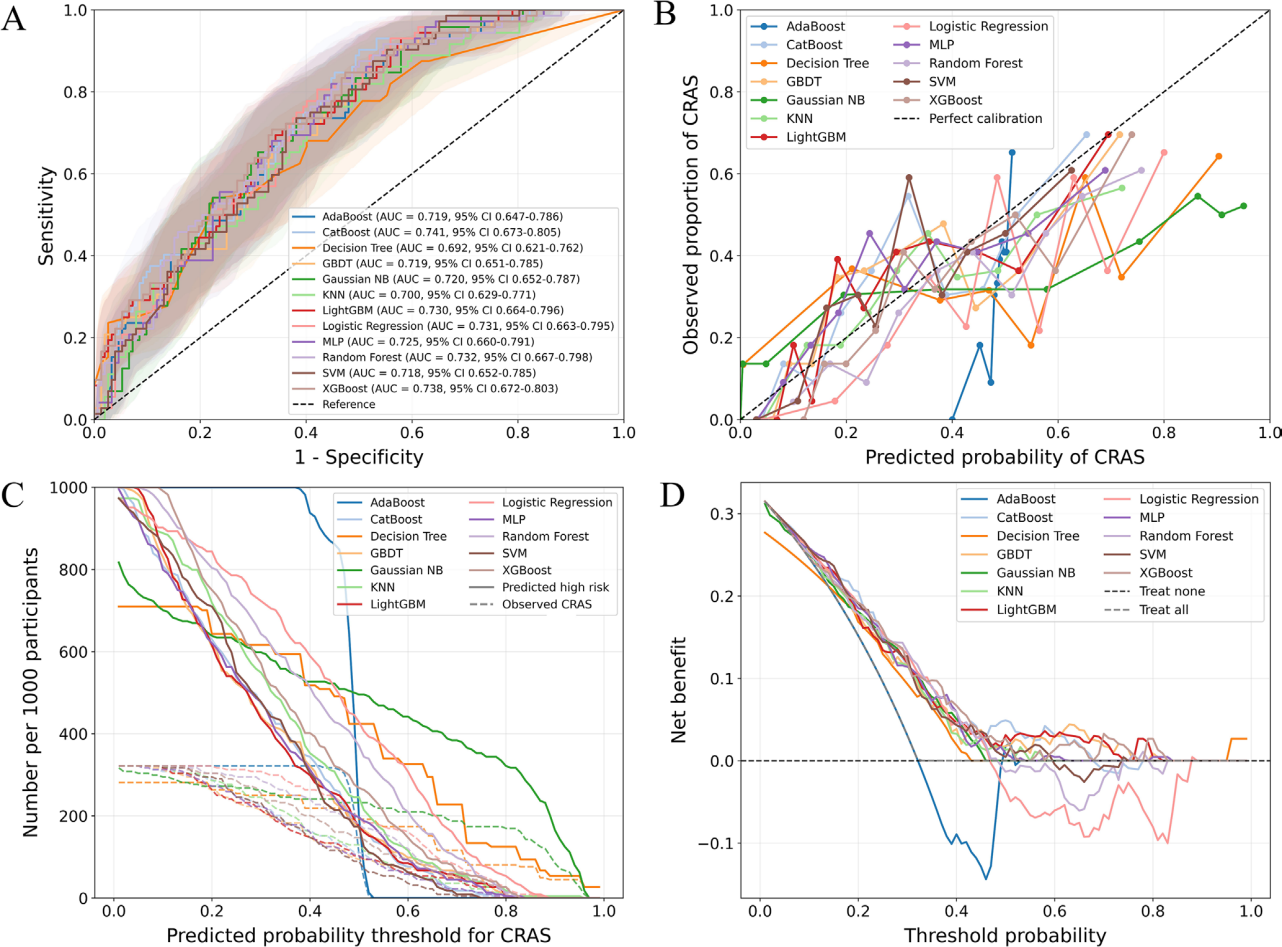
Predictive performance and clinical utility of 12 machine‐learning models in the NHANES internal validation set. (A) Receiver operating characteristic curves with bootstrap 95% confidence bands. (B) Calibration curves. (C) Clinical impact curves. (D) Decision curve analyses.

**FIGURE 8:**
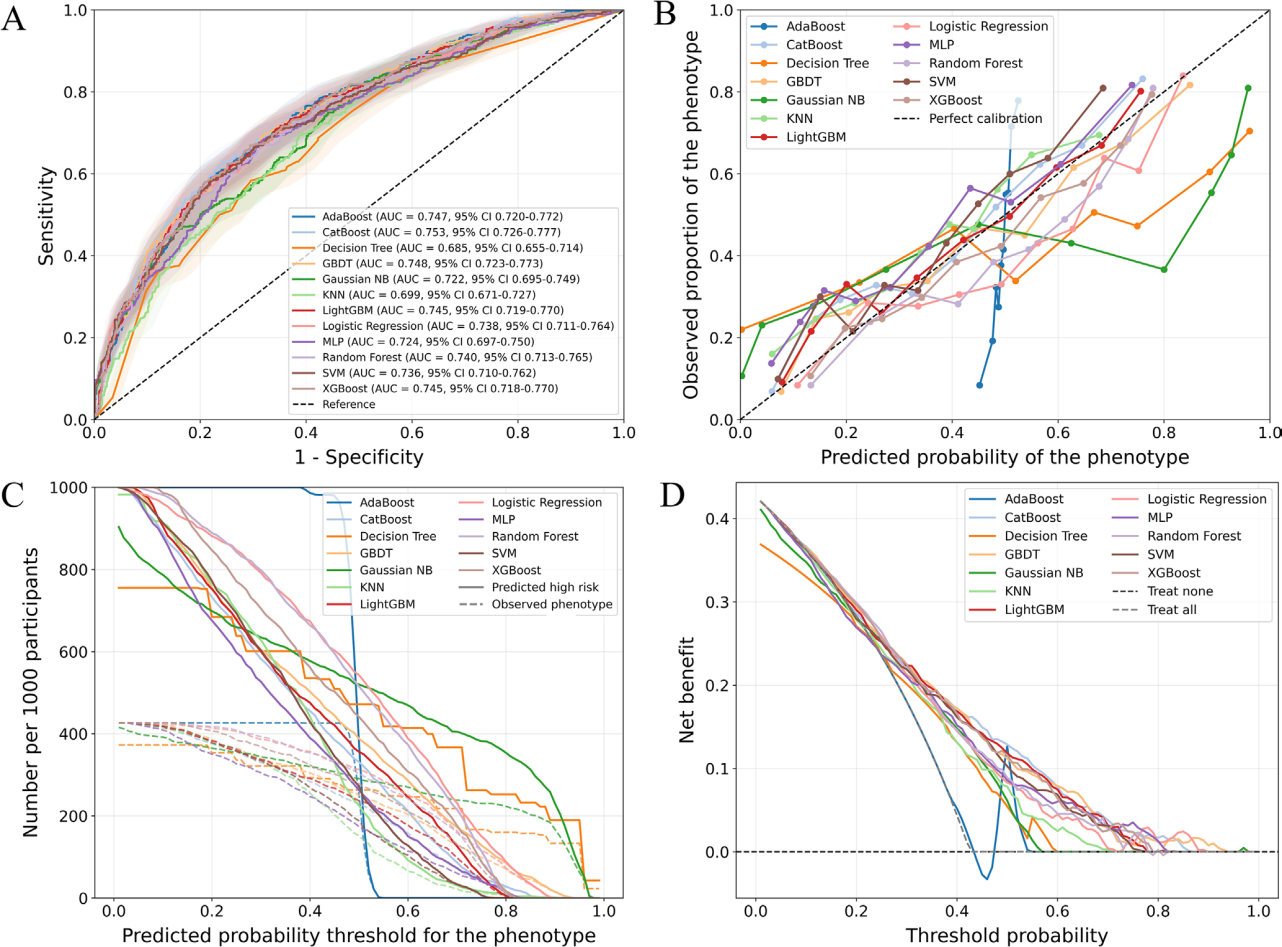
Exploratory cross‐phenotype application of 12 machine‐learning models to the MI–CKD–anemia phenotype in KNHANES. (A) Receiver operating characteristic curves with bootstrap 95% confidence bands. (B) Calibration curves. (C) Clinical impact curves. (D) Decision curve analyses.

ROC curves with bootstrap confidence bands, calibration curves, clinical impact curves, and decision curve analyses for the NHANES internal validation set and the exploratory KNHANES cross‐phenotype application are shown in Figures 7 and 8. CatBoost showed the highest ROC‐AUC in both analytic settings. Precision–recall curves are presented in Figure S3. The PR‐AUC values exceeded the corresponding outcome prevalences for the leading models in both settings, indicating discrimination beyond the prevalence‐based reference level. Calibration varied across algorithms; CatBoost had the lowest Brier score in both the NHANES internal validation set (0.1856) and the KNHANES cross‐phenotype application (0.2000).

The CIC visualized the expected number of participants classified as positive by the model and the corresponding number of true‐positive cases across a range of threshold probabilities. DCA quantified clinical usefulness by comparing model‐based net benefit with the “treat all” and “treat none” strategies. The magnitude of net benefit varied across models and threshold ranges in both analytic settings, underscoring the importance of interpreting clinical utility together with discrimination and calibration rather than relying on a single performance measure.

**FIGURE 9:**
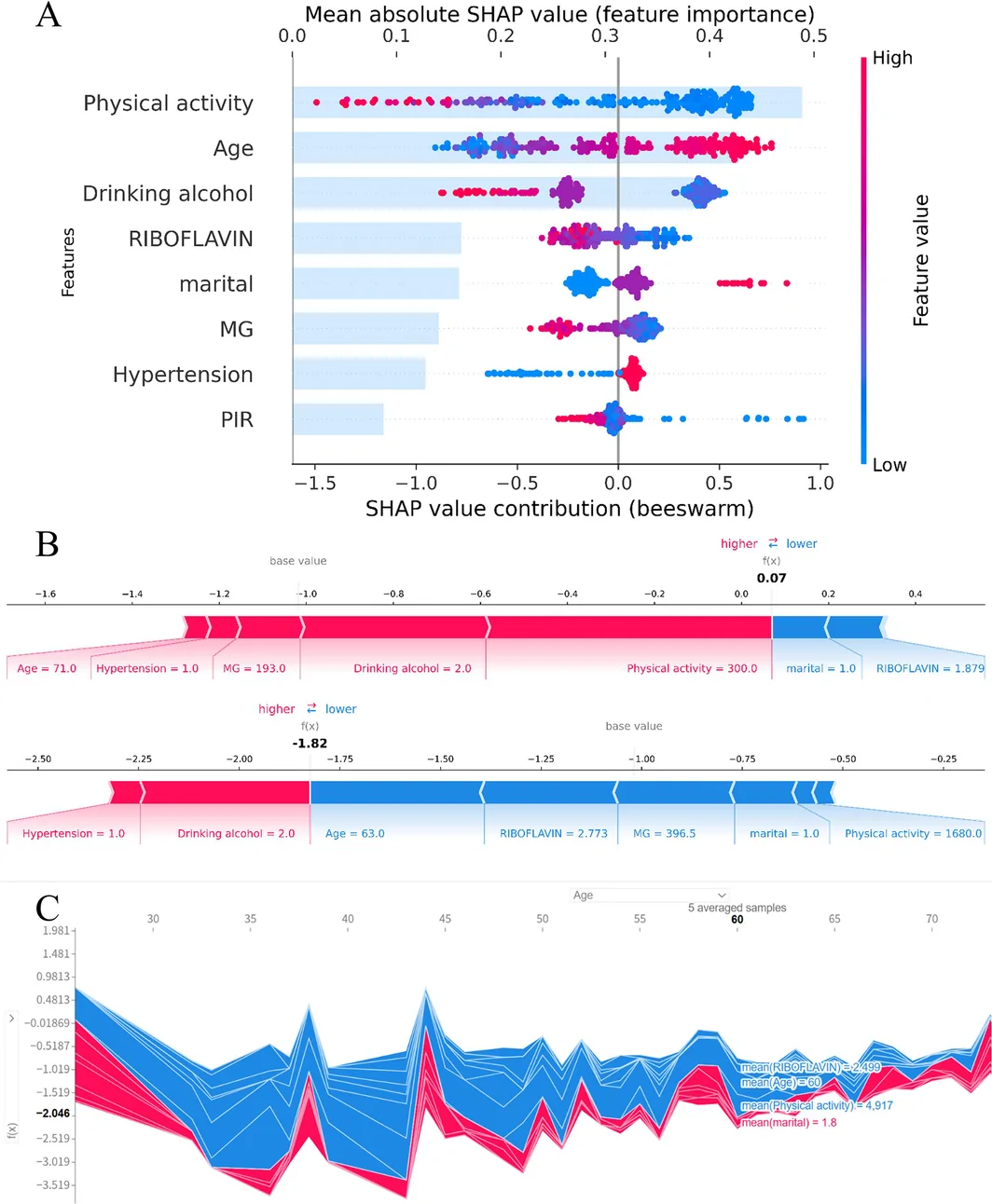
Interpretation of the CatBoost model using Shapley additive explanations (SHAP). (A) Combined SHAP beeswarm and feature importance summary plots showing the relative contribution and direction of each feature. (B) SHAP force plots for two representative patients. (C) SHAP stacked plot displaying the superimposed force plots.

Figure 9 presents the interpretability results for CatBoost in the NHANES internal validation set. The combined SHAP beeswarm and mean absolute SHAP summary plot in Figure 9A ranked physical activity, age, and drinking alcohol as the three most influential predictors, followed by riboflavin intake, marital status, magnesium intake, hypertension, and PIR. As shown in Table S15, these rankings were highly stable in the participant‐level bootstrap analysis: physical activity, age, and drinking alcohol retained median ranks of 1, 2, and 3, respectively, and each remained among the three highest‐ranked features in 100% of the 1000 bootstrap resamples. Higher age was generally associated with positive SHAP contributions, whereas higher magnesium and riboflavin intakes tended to be associated with lower predicted probability of CRAS. Figure 9B presents force plots for representative true‐positive and true‐negative participants. In these plots, red features shift the model output upward and blue features shift it downward; *f*(*x*) represents the model output for the individual participant relative to the baseline value. Figure 9C shows the stacked force plot ordered by participants' original age. The predicted probability of CRAS generally tended to increase with age ordering, consistent with the positive contribution of age observed in the SHAP summary.

## 4 Discussion

This study evaluated the association between DII and HF‐defined CRAS in NHANES and complemented it with an exploratory analysis of the MI–CKD–anemia phenotype in KNHANES. Higher DII was associated with greater odds of CRAS in NHANES, with a directionally similar association observed for the MI–CKD–anemia phenotype in KNHANES.

All six inflammatory indices showed positive indirect associations in NHANES, although these cross‐sectional estimates do not establish causal mediation. Among participants with HF, CatBoost had the highest ROC‐AUC in the NHANES internal validation set and was subsequently applied to the related KNHANES phenotype. It outperformed the age‐only model, whereas improvements over the conventional clinical model were smaller. SHAP analysis consistently identified physical activity, age, and alcohol use as the leading predictors.

The largely linear association in NHANES and nonlinear pattern in KNHANES suggest that the dose–response relationship may differ across populations and outcome definitions. Recalculating NHANES DII with the 22 components available in KNHANES yielded nearly identical estimates and the same overall pattern. The high correlation and quartile agreement between the 22‐ and 27‐component scores further suggest that component availability did not materially alter the NHANES findings.

These findings are consistent with previous studies linking higher DII to cardiovascular disease and cardiometabolic factors (Hariharan et al. 2022). Pro‐inflammatory diets have also been associated with adverse nutritional and muscle outcomes in CKD (Mansouri et al. 2024) and with a higher prevalence of anemia among people with diabetes (Cao et al. 2025). Together, this evidence supports further study of diet‐related inflammation across the cardiac, renal, and hematologic components of CRAS.

The exploratory mediation analyses indicated that all six inflammatory indices statistically accounted for part of the DII–CRAS association. IBI and CALLY had the largest estimated proportions. Both combine markers of inflammation, nutritional status, and immune balance, which may explain their stronger statistical contribution (Kingma et al. 2017; Xu et al. 2024). The SII estimate remained similar across sensitivity analyses. However, temporal ordering cannot be established in these cross‐sectional data, and the estimates may be affected by nonlinear relationships or covariate adjustment. The findings should therefore be interpreted as exploratory rather than as evidence of specific biological pathways.

The positive DII–CRAS association was supported by sensitivity analyses in NHANES, and subgroup estimates were generally consistent in direction across both cohorts. Interactions for education in NHANES and hypertension in KNHANES met the nominal significance threshold, but neither remained significant after FDR correction. These subgroup patterns may warrant further study but do not provide firm evidence of effect modification.

Among the 12 algorithms, CatBoost had the highest ROC‐AUC and lowest Brier score in the NHANES internal validation set. It outperformed the age‐only model in both evaluation settings, whereas its gains over the conventional clinical model were smaller. Its performance in KNHANES suggests that the selected predictors also contained information relevant to the related MI–CKD–anemia phenotype, but this application was not a direct validation of HF‐defined CRAS. PR‐AUC provided additional information in the presence of class imbalance, whereas the Brier score summarized probabilistic accuracy.

SHAP analysis consistently ranked physical activity, age, and alcohol use as the three leading predictors. Magnesium and riboflavin were the only nutrient‐intake variables retained in the final model and generally contributed to lower predicted probabilities of CRAS.

Among the retained nutrient predictors, higher magnesium and riboflavin intakes were generally associated with lower SHAP values and lower predicted probability of CRAS. Magnesium is involved in enzymatic activity, ion‐channel regulation, inflammatory signaling, and pathways relevant to cardiovascular and renal injury (Ashique et al. 2023; Long et al. 2022; Liu and Dudley 2020). Riboflavin is essential for cellular redox reactions and may also support iron mobilization, hemoglobin synthesis, and hematologic function (Ashoori and Saedisomeolia 2014; Powers 2003; Aljaadi et al. 2022). These functions are consistent with the patterns observed in the fitted model.

The study has several strengths. NHANES and KNHANES provided large, nationally representative samples for the primary CRAS analysis and a complementary analysis of a related phenotype. We evaluated six inflammatory indices and tested the robustness of the main association in several sensitivity analyses. The prediction analysis compared 12 algorithms with clinical benchmark models and used SHAP to examine feature contributions and ranking stability.

Nevertheless, several limitations should be considered. Both surveys are cross‐sectional, so the temporal order of diet, inflammation, and CRAS cannot be established. Reverse causation is possible if participants changed their diets after a diagnosis of HF, CKD, or anemia. The mediation results may also be affected by nonlinearity, residual confounding, overadjustment, or collider bias. Despite FDR correction within related test families, false‐positive findings remain possible, particularly in secondary and subgroup analyses.

HF was identified by self‐reported physician diagnosis rather than clinical adjudication. Recall error, underdiagnosis, and differences in HF phenotype or severity may therefore have caused misclassification. This could also affect the target population for machine learning, model calibration, performance estimates, and SHAP rankings.

KNHANES evaluated an MI–CKD–anemia phenotype rather than HF‐defined CRAS. Differences between the surveys in diet, BMI, comorbidities, healthcare settings, and outcome prevalence may have affected predictor distributions and model calibration. Standardization and variable harmonization ensured consistent model inputs but could not remove these population differences. The models were applied without recalibration; the KNHANES findings should therefore be regarded as exploratory cross‐phenotype results rather than external validation. Independent cohorts with a consistent CRAS definition are needed before clinical use.

Although no synthetic oversampling was used and model development incorporated nested cross‐validation, an untouched internal validation set, bootstrap confidence intervals, and participant‐level SHAP stability analyses, the number of CRAS cases available for model development remained modest. In addition, the final algorithm was selected and its performance was reported using the same internal validation set, which may introduce some optimism into the performance estimate. The SHAP bootstrap analysis assessed ranking stability conditional on the fixed fitted model and did not capture variability arising from repeated feature selection or model refitting. Accordingly, the reported predictive performance and feature‐importance rankings should be interpreted cautiously pending evaluation in additional clinically characterized cohorts. In addition, the CRAS components and inflammatory indices were derived from single‐time laboratory measurements, which may not fully capture long‐term inflammatory or nutritional status.

DII was calculated from 24‐h dietary recalls and may be affected by reporting error and day‐to‐day variation. The limited number of recalls may not fully represent habitual intake. Although the NHANES results were similar with the 22‐ and 27‐component scores, differences in available dietary components may still limit direct comparison of absolute DII values and per‐unit estimates between surveys. The difference in DII components did not directly affect the KNHANES model application, as total DII was not retained and the selected nutrient predictors were available in both datasets. Residual confounding remains possible, and restricting model predictors to variables harmonized across the surveys may have limited model performance and clinical completeness. Despite these limitations, we believe our study contributes to understanding the relationship between dietary inflammatory potential patterns and CRAS and the possible statistical contribution of systemic inflammatory indices. The prediction model may assist clinicians in earlier identification of CRAS and provide insights for prognostic research.

## 5 Conclusion

Higher DII was associated with greater odds of HF‐defined CRAS in NHANES, with a similar direction of association for the related MI–CKD–anemia phenotype in KNHANES. Exploratory mediation analyses suggested that systemic inflammatory indices may partly explain the DII–CRAS association in NHANES. Among participants with heart failure, we developed and compared machine‐learning models for CRAS prediction, and their exploratory application to the MI–CKD–anemia phenotype in KNHANES provided preliminary information on cross‐phenotype performance.

## Author Contributions

**Xiaoxiangxin Luo:** methodology, writing – original draft. **Wenjun Ding:** methodology, writing – original draft. **Lihan Wu:** formal analysis, validation. **Yadan Chen:** conceptualization, validation, writing – original draft, writing – review and editing. **Shirong Lin:** conceptualization, methodology, data curation, writing – original draft. **Haiyang Song:** supervision, methodology. **Weijia Zeng:** investigation, formal analysis. **Yufeng Chen:** conceptualization, methodology, data curation, writing – original draft. **Jun Ke:** methodology, data curation. **Feng Chen:** project administration, supervision.

## Funding

This work was supported by Joint Funds for the Innovation of Science and Technology, Fujian Province (Grant number: 2024Y9036) and Fujian Provincial Health Technology Project (Grant number: 2022ZD01008). The funders had no role in the study design, data collection, analysis, interpretation, or manuscript writing.

## Ethics Statement

This study is a secondary analysis of de‐identified, publicly available data from the National Health and Nutrition Examination Survey (NHANES) and the Korea National Health and Nutrition Examination Survey (KNHANES). The NHANES study protocols were approved by the National Center for Health Statistics (NCHS) Research Ethics Review Board, and written informed consent was obtained from all participants. KNHANES is conducted by the Korea Disease Control and Prevention Agency (KDCA) and approved by the KDCA Institutional Review Board (IRB), with written informed consent obtained from all participants. The study was conducted in accordance with the Declaration of Helsinki. The present analysis used only anonymized public‐use datasets; therefore, no additional ethics approval or consent was required.

## Conflicts of Interest

The authors declare no conflicts of interest.

## Supporting information


**Table S1:** Overall inflammatory effect score for 27 specific food parameters.
**Table S2:** Definitions of variables.
**Figure S1:** Data distribution and skewness characteristics of the six inflammatory indices. (A) Histograms of the six inflammatory indices. (B) Box plots of the six inflammatory indices.
**Table S3:** Variable‐specific missingness and missingness patterns before KNN imputation.
**Table S4:** Sensitivity analyses of the indirect association through SII in the association between DII and CRAS.
**Table S5:** Benjamini–Hochberg false discovery rate correction for exploratory analyses.
**Table S6:** Hyperparameter search spaces and final selected settings for the 12 machine‐learning algorithms.
**Table S7:** Baseline characteristics of participants stratified by Dietary Inflammatory Index quartiles in KNHANES.
**Table S8:** Baseline characteristic table based on CRAS grouping in NHANES.
**Table S9:** Baseline characteristics by MI–CKD–anemia phenotype in the KNHANES myocardial infarction subgroup.
**Table S10:** The associations between Six Systemic Inflammatory Indices and CRAS in weighted logistic regression models.
**Table S11:** Sensitivity analysis of the association between DII and CRAS.
**Table S12:** Sensitivity analysis of the association between the shared 22‐component Dietary Inflammatory Index and CRAS in NHANES.
**Figure S2:** Restricted cubic spline analysis of the association between the shared 22‐component DII and CRAS in NHANES.
**Figure S3:** Precision–recall curves for the 12 machine‐learning models. (A) Prediction of HF‐defined CRAS in the NHANES internal validation set. (B) Exploratory cross‐phenotype application to the MI–CKD–anemia phenotype in KNHANES. The horizontal dashed line in each panel represents the observed outcome prevalence in the corresponding evaluation sample. AP denotes average precision.
**Table S13:** Performance of CatBoost and clinically defined benchmark models in the NHANES internal validation set and KNHANES cross‐phenotype application.
**Table S14:** Paired differences in predictive performance between CatBoost and the clinically defined benchmark models.
**Figure S4:** Receiver operating characteristic and precision–recall curves comparing CatBoost with the age‐only and conventional clinical logistic regression models. (A) ROC curves in the NHANES internal validation set; (B) precision–recall curves in the NHANES internal validation set; (C) ROC curves in the KNHANES cross‐phenotype application; and (D) precision–recall curves in the KNHANES cross‐phenotype application.
**Table S15:** Bootstrap stability of global SHAP feature‐importance rankings for the CatBoost model in the NHANES internal validation set.

## Data Availability

The data analyzed in this study are publicly available from the National Health and Nutrition Examination Survey (NHANES): https://www.cdc.gov/nchs/nhanes/, and the Korea National Health and Nutrition Examination Survey (KNHANES): https://knhanes.kdca.go.kr/knhanes/, further inquiries can be directed to the corresponding author.

## References

[fsn372232-bib-0001] Ahluwalia, N. , J. Dwyer , A. Terry , A. Moshfegh , and C. Johnson . 2016. “Update on NHANES Dietary Data: Focus on Collection, Release, Analytical Considerations, and Uses to Inform Public Policy.” Advances in Nutrition 7, no. 1: 121–134. 10.3945/an.115.009258. 26773020 PMC4717880

[fsn372232-bib-0002] Aljaadi, A. M. , A. M. Devlin , and T. J. Green . 2022. “Riboflavin Intake and Status and Relationship to Anemia.” Nutrition Reviews 81, no. 1: 114–132. 10.1093/nutrit/nuac043. 36018769

[fsn372232-bib-0003] Altamimi, A. , A. A. Alarfaj , M. Umer , et al. 2024. “An Automated Approach to Predict Diabetic Patients Using KNN Imputation and Effective Data Mining Techniques.” BMC Medical Research Methodology 24, no. 1: 221. 10.1186/s12874-024-02324-0. 39333904 PMC11438170

[fsn372232-bib-0004] Ashique, S. , S. Kumar , A. Hussain , et al. 2023. “A Narrative Review on the Role of Magnesium in Immune Regulation, Inflammation, Infectious Diseases, and Cancer.” Journal of Health, Population and Nutrition 42, no. 1: 74. 10.1186/s41043-023-00423-0. 37501216 PMC10375690

[fsn372232-bib-0005] Ashoori, M. , and A. Saedisomeolia . 2014. “Riboflavin (Vitamin B2) and Oxidative Stress: A Review.” British Journal of Nutrition 111, no. 11: 1985–1991. 10.1017/S0007114514000178. 24650639

[fsn372232-bib-0006] Cao, N. , J. Li , C. Ling , J. Wang , and F. An . 2025. “The Association Between Dietary Inflammatory Index and Anemia in Individuals With Diabetes Mellitus.” Frontiers in Nutrition 12: 1538696. 10.3389/fnut.2025.1538696. 40034738 PMC11874837

[fsn372232-bib-0007] Gauthier, J. , Q. V. Wu , and T. A. Gooley . 2020. “Cubic Splines to Model Relationships Between Continuous Variables and Outcomes: A Guide for Clinicians.” Bone Marrow Transplantation 55, no. 4: 675–680. 10.1038/s41409-019-0679-x. 31576022

[fsn372232-bib-0008] Guan, C. , A. Gong , Y. Zhao , et al. 2024. “Interpretable Machine Learning Model for New‐Onset Atrial Fibrillation Prediction in Critically Ill Patients: A Multi‐Center Study.” Critical Care 28, no. 1: 349. 10.1186/s13054-024-05138-0. 39473013 PMC11523862

[fsn372232-bib-0009] Hariharan, R. , E. N. Odjidja , D. Scott , et al. 2022. “The Dietary Inflammatory Index, Obesity, Type 2 Diabetes, and Cardiovascular Risk Factors and Diseases.” Obesity Reviews 23, no. 1: e13349. 10.1111/obr.13349. 34708499

[fsn372232-bib-0010] Hébert, J. R. , N. Shivappa , M. D. Wirth , J. R. Hussey , and T. G. Hurley . 2019. “Perspective: The Dietary Inflammatory Index (DII)—Lessons Learned, Improvements Made, and Future Directions.” Advances in Nutrition 10, no. 2: 185–195. 10.1093/advances/nmy071. 30615051 PMC6416047

[fsn372232-bib-0011] Hu, S. , J. Song , H. Jiang , B. Wei , and H. Wang . 2025. “Association Between the Dietary Index for Gut Microbiota and Cardiometabolic Multimorbidity: Systemic Immune‐Inflammation Index and Systemic Inflammatory Response Index.” Frontiers in Nutrition 12: 1591799. 10.3389/fnut.2025.1591799. 40538590 PMC12176575

[fsn372232-bib-0012] Huang, Q. , J. Wan , W. Nan , S. Li , B. He , and Z. Peng . 2024. “Association Between Manganese Exposure in Heavy Metals Mixtures and the Prevalence of Sarcopenia in US Adults From NHANES 2011–2018.” Journal of Hazardous Materials 464: 133005. 10.1016/j.jhazmat.2023.133005. 37988867

[fsn372232-bib-0013] Islam, M. M. , M. O. Satici , and S. E. Eroglu . 2024. “Unraveling the Clinical Significance and Prognostic Value of the Neutrophil‐to‐Lymphocyte Ratio, Platelet‐to‐Lymphocyte Ratio, Systemic Immune‐Inflammation Index, Systemic Inflammation Response Index, and Delta Neutrophil Index: An Extensive Literature Review.” Turkish Journal of Emergency Medicine 24, no. 1: 8–19. 10.4103/tjem.tjem_198_23. 38343523 PMC10852137

[fsn372232-bib-0014] Kadatane, S. P. , M. Satariano , M. Massey , K. Mongan , and R. Raina . 2023. “The Role of Inflammation in CKD.” Cells 12, no. 12: 1581. 10.3390/cells12121581. 37371050 PMC10296717

[fsn372232-bib-0015] Kerr, K. F. , M. D. Brown , K. Zhu , and H. Janes . 2016. “Assessing the Clinical Impact of Risk Prediction Models With Decision Curves: Guidance for Correct Interpretation and Appropriate Use.” Journal of Clinical Oncology 34, no. 21: 2534–2540. 10.1200/JCO.2015.65.5654. 27247223 PMC4962736

[fsn372232-bib-0016] Kiecolt‐Glaser, J. K. , M. A. Belury , R. Andridge , W. B. Malarkey , B. S. Hwang , and R. Glaser . 2012. “Omega‐3 Supplementation Lowers Inflammation in Healthy Middle‐Aged and Older Adults: A Randomized Controlled Trial.” Brain, Behavior, and Immunity 26, no. 6: 988–995. 10.1016/j.bbi.2012.05.011. 22640930 PMC3398219

[fsn372232-bib-0017] Kingma, J. G. , D. Simard , J. R. Rouleau , B. Drolet , and C. Simard . 2017. “The Physiopathology of Cardiorenal Syndrome: A Review of the Potential Contributions of Inflammation.” Journal of Cardiovascular Development and Disease 4, no. 4: 21. 10.3390/jcdd4040021. 29367550 PMC5753122

[fsn372232-bib-0018] Kweon, S. , Y. Kim , M. J. Jang , et al. 2014. “Data Resource Profile: The Korea National Health and Nutrition Examination Survey (KNHANES).” International Journal of Epidemiology 43, no. 1: 69–77. 10.1093/ije/dyt228. 24585853 PMC3937975

[fsn372232-bib-0019] Levey, A. S. , L. A. Stevens , C. H. Schmid , et al. 2009. “A New Equation to Estimate Glomerular Filtration Rate.” Annals of Internal Medicine 150, no. 9: 604–612. 10.7326/0003-4819-150-9-200905050-00006. 19414839 PMC2763564

[fsn372232-bib-0020] Liu, B. , J. Wang , Y. Li , K. Li , and Q. Zhang . 2023. “The Association Between Systemic Immune‐Inflammation Index and Rheumatoid Arthritis: Evidence From NHANES 1999–2018.” Arthritis Research & Therapy 25, no. 1: 34. 10.1186/s13075-023-03018-6. 36871051 PMC9985219

[fsn372232-bib-0021] Liu, J. , K. Chen , M. Tang , et al. 2025. “Oxidative Stress and Inflammation Mediate the Adverse Effects of Cadmium Exposure on All‐Cause and Cause‐Specific Mortality in Patients With Diabetes and Prediabetes.” Cardiovascular Diabetology 24, no. 1: 145. 10.1186/s12933-025-02698-5. 40158078 PMC11954339

[fsn372232-bib-0022] Liu, M. , and S. C. Dudley . 2020. “Magnesium, Oxidative Stress, Inflammation, and Cardiovascular Disease.” Antioxidants 9, no. 10: 907. 10.3390/antiox9100907. 32977544 PMC7598282

[fsn372232-bib-0023] Long, M. , X. Zhu , X. Wei , et al. 2022. “Magnesium in Renal Fibrosis.” International Urology and Nephrology 54, no. 8: 1881–1889. 10.1007/s11255-022-03118-3. 35060008

[fsn372232-bib-0024] Ma, F. , L. Li , L. Xu , et al. 2023. “The Relationship Between Systemic Inflammation Index, Systemic Immune‐Inflammatory Index, and Inflammatory Prognostic Index and 90‐Day Outcomes in Acute Ischemic Stroke Patients Treated With Intravenous Thrombolysis.” Journal of Neuroinflammation 20, no. 1: 220. 10.1186/s12974-023-02890-y. 37777768 PMC10543872

[fsn372232-bib-0025] Ma, R. , L. Cui , J. Cai , et al. 2024. “Association Between Systemic Immune Inflammation Index, Systemic Inflammation Response Index and Adult Psoriasis: Evidence From NHANES.” Frontiers in Immunology 15: 1323174. 10.3389/fimmu.2024.1323174. 38415255 PMC10896999

[fsn372232-bib-0026] Manla, Y. , O. Kholoki , F. Bader , et al. 2023. “The Prevalence of Cardiorenal Anemia Syndrome Among Patients With Heart Failure and Its Association With All‐Cause Hospitalizations: A Retrospective Single‐Center Study From the Middle East.” Frontiers in Cardiovascular Medicine 10: 1244275. 10.3389/fcvm.2023.1244275. 37767373 PMC10520954

[fsn372232-bib-0027] Mann, D. L. 2015. “Innate Immunity and the Failing Heart: The Cytokine Hypothesis Revisited.” Circulation Research 116, no. 7: 1254–1268. 10.1161/CIRCRESAHA.116.302317. 25814686 PMC4380242

[fsn372232-bib-0028] Mansouri, F. , F. Jafari , S. Ranjbar , et al. 2024. “Dietary Inflammatory Index Could Increase the Risk of Sarcopenia in Patients With Chronic Kidney Disease.” Scientific Reports 14, no. 1: 15284. 10.1038/s41598-024-65340-6. 38961105 PMC11222548

[fsn372232-bib-0029] McCullough, P. A. 2021. “Anemia of Cardiorenal Syndrome.” Kidney International Supplements 11, no. 1: 35–45. 10.1016/j.kisu.2020.12.001. 33777494 PMC7983020

[fsn372232-bib-0030] Mwololo, C. M. 2013. “Anemia and Renal Dysfunction in Patients With Ambulatory Heart Failure at the Kenyatta National Hospital.” University of Nairobi, Master's thesis. http://hdl.handle.net/11295/63382.

[fsn372232-bib-0031] Naderinejad, N. , H.‐S. Ejtahed , G. Asghari , P. Mirmiran , and F. Azizi . 2020. “Dietary Patterns and Risk of Chronic Kidney Disease Among Tehranian Adults With High Blood Pressure.” International Journal of Endocrinology and Metabolism 18, no. 1: e89709. 10.5812/ijem.89709. 32308695 PMC7144243

[fsn372232-bib-0032] National Center for Health Statistics . 2020. National Health and Nutrition Examination Survey 2017–2018 Data Documentation, Codebook, and Frequencies: Medical Conditions (MCQ_J). Centers for Disease Control and Prevention. https://wwwn.cdc.gov/Nchs/Data/Nhanes/Public/2017/DataFiles/MCQ_J.htm.

[fsn372232-bib-0033] Nohara, Y. , K. Matsumoto , H. Soejima , and N. Nakashima . 2022. “Explanation of Machine Learning Models Using Shapley Additive Explanation and Application for Real Data in Hospital.” Computer Methods and Programs in Biomedicine 214: 106584. 10.1016/j.cmpb.2021.106584. 34942412

[fsn372232-bib-0034] Palazzuoli, A. , G. Antonelli , and R. Nuti . 2011. “Anemia in Cardio‐Renal Syndrome: Clinical Impact and Pathophysiologic Mechanisms.” Heart Failure Reviews 16, no. 6: 603–607. 10.1007/s10741-011-9230-x. 21336549

[fsn372232-bib-0035] Powers, H. J. 2003. “Riboflavin (Vitamin B‐2) and Health.” American Journal of Clinical Nutrition 77, no. 6: 1352–1360. 10.1093/ajcn/77.6.1352. 12791609

[fsn372232-bib-0036] Rahmani, A. M. , E. Yousefpoor , M. S. Yousefpoor , et al. 2021. “Machine Learning (ML) in Medicine: Review, Applications, and Challenges.” Mathematics 9, no. 22: 2970. 10.3390/math9222970.

[fsn372232-bib-0037] Rangaswami, J. , V. Bhalla , J. E. A. Blair , et al. 2019. “Cardiorenal Syndrome: Classification, Pathophysiology, Diagnosis, and Treatment Strategies: A Scientific Statement From the American Heart Association.” Circulation 139, no. 16: e840–e878. 10.1161/CIR.0000000000000664. 30852913

[fsn372232-bib-0038] Shehab, M. , L. Abualigah , Q. Shambour , et al. 2022. “Machine Learning in Medical Applications: A Review of State‐of‐the‐Art Methods.” Computers in Biology and Medicine 145: 105458. 10.1016/j.compbiomed.2022.105458. 35364311

[fsn372232-bib-0039] Shivappa, N. , J. R. Hebert , A. Marcos , et al. 2017. “Association Between Dietary Inflammatory Index and Inflammatory Markers in the HELENA Study.” Molecular Nutrition & Food Research 61, no. 6: 201600707. 10.1002/mnfr.201600707. PMC5517083 27981781

[fsn372232-bib-0040] Shivappa, N. , J. R. Hébert , E. R. Rietzschel , et al. 2015. “Associations Between Dietary Inflammatory Index and Inflammatory Markers in the Asklepios Study.” British Journal of Nutrition 113, no. 4: 665–671. 10.1017/S000711451400395X. 25639781 PMC4355619

[fsn372232-bib-0041] Shivappa, N. , S. E. Steck , T. G. Hurley , J. R. Hussey , and J. R. Hébert . 2014. “Designing and Developing a Literature‐Derived, Population‐Based Dietary Inflammatory Index.” Public Health Nutrition 17, no. 8: 1689–1696. 10.1017/S1368980013002115. 23941862 PMC3925198

[fsn372232-bib-0042] Shu, Y. , X. Wu , J. Wang , X. Ma , H. Li , and Y. Xiang . 2022. “Associations of Dietary Inflammatory Index With Prediabetes and Insulin Resistance.” Frontiers in Endocrinology 13: 820932. 10.3389/fendo.2022.820932. 35250879 PMC8892213

[fsn372232-bib-0043] Silverberg, D. S. , D. Wexler , M. Blum , et al. 2000. “The Use of Subcutaneous Erythropoietin and Intravenous Iron for the Treatment of the Anemia of Severe, Resistant Congestive Heart Failure Improves Cardiac and Renal Function and Functional Cardiac Class, and Markedly Reduces Hospitalizations.” Journal of the American College of Cardiology 35, no. 7: 1737–1744. 10.1016/S0735-1097(00)00613-6. 10841219

[fsn372232-bib-0044] Silverberg, D. S. , D. Wexler , and A. Iaina . 2004. “The Role of Anemia in Congestive Heart Failure and Chronic Kidney Insufficiency: The Cardio Renal Anemia Syndrome.” Perspectives in Biology and Medicine 47, no. 4: 575–589. 10.1353/pbm.2004.0072. 15467179

[fsn372232-bib-0045] Stevens, P. E. , S. B. Ahmed , J. J. Carrero , et al. 2024. “KDIGO 2024 Clinical Practice Guideline for the Evaluation and Management of Chronic Kidney Disease.” Kidney International 105, no. 4S: S117–S314. 10.1016/j.kint.2023.10.018. 38490803

[fsn372232-bib-0046] Tuzimek, A. , E. A. Dziedzic , J. Beck , and W. Kochman . 2024. “Correlations Between Acute Coronary Syndrome and Novel Inflammatory Markers (Systemic Immune‐Inflammation Index, Systemic Inflammation Response Index, and Aggregate Index of Systemic Inflammation) in Patients With and Without Diabetes or Prediabetes.” Journal of Inflammation Research 17: 2623–2632. 10.2147/JIR.S454117. 38707954 PMC11067916

[fsn372232-bib-0047] Wirth, M. D. , J. R. Hébert , N. Shivappa , et al. 2016. “Anti‐Inflammatory Dietary Inflammatory Index Scores Are Associated With Healthier Scores on Other Dietary Indices.” Nutrition Research 36, no. 3: 214–219. 10.1016/j.nutres.2015.11.009. 26923507 PMC4773655

[fsn372232-bib-0048] World Health Organization . 2011. Haemoglobin Concentrations for the Diagnosis of Anaemia and Assessment of Severity. World Health Organization. https://www.who.int/publications/i/item/WHO‐NMH‐NHD‐MNM‐11.1.

[fsn372232-bib-0049] Wu, B. , J. Liu , C. Shao , D. Yu , and J. Liao . 2025. “Integrating Inflammation, Nutrition, and Immunity: The CALLY Index as a Prognostic Tool in Digestive System Cancers – A Systematic Review and Meta‐Analysis.” BMC Cancer 25, no. 1: 672. 10.1186/s12885-025-14074-3. 40217204 PMC11992890

[fsn372232-bib-0050] Xu, Z. , J. Tang , X. Chen , Y. Jin , H. Zhang , and R. Liang . 2024. “Associations of c‐Reactive Protein‐Albumin‐Lymphocyte (CALLY) Index With Cardiorenal Syndrome: Insights From a Population‐Based Study.” Heliyon 10, no. 17: e37197. 10.1016/j.heliyon.2024.e37197. 39296012 PMC11408039

[fsn372232-bib-0051] Zhang, Z. , and H. Ni . 2011. “C‐Reactive Protein as a Predictor of Mortality in Critically Ill Patients: A Meta‐Analysis and Systematic Review.” Anaesthesia and Intensive Care 39, no. 5: 854–861. 10.1177/0310057X1103900509. 21970129

