# Supplementary material for: Association Between the Dietary Inflammatory Index and Cardiorenal Anemia Syndrome: Evidence From NHANES and Related‐Phenotype Exploration in KNHANES
Source: Food Sci Nutr. 2026 Sep 2;14(9):e72232. doi: 10.1002/fsn3.72232 (PMC13536486; doi:10.1002/fsn3.72232)
Supplement: Supplementary file 1 [file FSN3-14-e72232-s001.docx]

**Table S1** Overall Inflammatory Effect Score for 27 Specific Food Parameters

| **Food** | **Overall inflammatory effect score** |
| --- | --- |
| Alcohol (g) | −0.278 |
| Vitamin B12 (μg) | 0.106 |
| Vitamin B6 (mg) | −0.365 |
| β-Carotene (μg) | −0.584 |
| Caffeine (g) | −0.110 |
| Carbohydrate (g) | 0.097 |
| Cholesterol (mg) | 0.110 |
| Energy (kcal) | 0.180 |
| Total fat (g) | 0.298 |
| Fibre (g) | −0.663 |
| Folic acid (μg) | −0.190 |
| Fe (mg) | 0.032 |
| Mg (mg) | −0.484 |
| MUFA (g) | −0.009 |
| Niacin (mg) | −0.246 |
| *n*-3 Fatty acids (g)  *n*-6 Fatty acids (g) | −0.436  −0.159 |
| Protein (g) | 0.021 |
| PUFA (g) | −0.337 |
| Riboflavin (mg) | −0.068 |
| Saturated fat (g) | 0.373 |
| Se (μg) | −0.191 |
| Thiamin (mg) | −0.098 |
| Vitamin A (RE) | −0.401 |
| Vitamin C (mg) | −0.424 |
| Vitamin E (mg) | −0.419 |
| Zn (mg) | −0.313 |

**Table S2** Definitions of Variables

| **Category** | **Variable** | **Definition** |
| --- | --- | --- |
| **Demographic characteristics** | Age | Continuous variable, measured in years. |
|  | Sex | Male; Female. |
|  | Ethnicity | Non-Hispanic White; Non-Hispanic Black; Mexican American; Other Hispanic; Other Race (including multi-racial). |
|  | Marital status | Married/Living with partner; Widowed/Divorced/Separated; Never married. |
|  | Poverty Income Ratio(PIR) | Ratio of family income to the federal poverty threshold, indicator of socioeconomic status. |
|  | Education | Below high school; High school; Above high school. |
| **Lifestyle factors** | Smoking status | Never smoker; Former smoker; Current smoker. |
|  | Drinking alcohol | Never; Former; Mild; Moderate; Heavy. |
|  | Physical activity (PA_total_MET) | Total metabolic equivalent (MET) minutes per week, calculated from self-reported physical activity. |
|  | BMI (Body Mass Index) | Calculated as weight (kg) divided by height (m)². |
| **Comorbidities** | Hypertension | Defined as mean SBP ≥140 mmHg or DBP ≥90 mmHg (based on ≥3 measurements, or single measurement if only one available), or use of antihypertensive drugs, or physician diagnosis, or self-reported antihypertensive treatment. |
|  | Diabetes Mellitus (DM) | Physician diagnosis, or HbA1c ≥6.5%, or fasting glucose ≥7.0 mmol/L, or random glucose ≥11.1 mmol/L, or OGTT 2-h glucose ≥11.1 mmol/L, or current use of antidiabetic drugs/insulin. Subtypes: IFG (fasting glucose 6.1–6.9 mmol/L); IGT (2-h OGTT glucose 7.8–11.0 mmol/L). |
|  | Hyperlipidemia | Total cholesterol ≥200 mg/dL; or LDL-C ≥130 mg/dL; or HDL-C <40 mg/dL (men) / <50 mg/dL (women); or triglycerides ≥150 mg/dL; or current use of lipid-lowering drugs; or physician diagnosis/self-report of hyperlipidemia. |

**Figure S1** Data distribution and skewness characteristics of the six inflammatory indices. (A) Histograms of the six inflammatory indices. (B) Box plots of the six inflammatory indices.

**Table S3** Variable-specific missingness and missingness patterns before KNN imputation

| **Panel A. Variable-specific missingness** |  |  |  |  |  |  |
| --- | --- | --- | --- | --- | --- | --- |
| **Cohort** | **Variable** | **Total n** | **Available n** | **Missing n** | **Missing, %** | **Missing-data handling** |
| NHANES | Age | 25,388 | 25,388 | 0 | 0 | No imputation required |
| NHANES | Sex | 25,388 | 25,388 | 0 | 0 | No imputation required |
| NHANES | Ethnicity | 25,388 | 25,388 | 0 | 0 | No imputation required |
| NHANES | Marital status | 25,388 | 24,898 | 490 | 1.93 | KNN imputation |
| NHANES | PIR | 25,388 | 23,627 | 1,761 | 6.94 | KNN imputation |
| NHANES | Education | 25,388 | 24,883 | 505 | 1.99 | KNN imputation |
| NHANES | Smoking status | 25,388 | 24,592 | 796 | 3.14 | KNN imputation |
| NHANES | Alcohol use | 25,388 | 24,412 | 976 | 3.84 | KNN imputation |
| NHANES | Physical activity | 25,388 | 23,954 | 1,434 | 5.65 | KNN imputation |
| NHANES | BMI | 25,388 | 24,609 | 779 | 3.07 | KNN imputation |
| NHANES | Hypertension | 25,388 | 24,928 | 460 | 1.81 | KNN imputation |
| NHANES | Diabetes mellitus | 25,388 | 24,828 | 560 | 2.21 | KNN imputation |
| NHANES | Hyperlipidemia | 25,388 | 24,742 | 646 | 2.54 | KNN imputation |
| KNHANES | Age | 32,935 | 32,935 | 0 | 0 | No imputation required |
| KNHANES | Sex | 32,935 | 32,935 | 0 | 0 | No imputation required |
|  |  |  |  |  |  |  |
| continued |  |  |  |  |  |  |
| **Cohort** | **Variable** | **Total n** | **Available n** | **Missing n** | **Missing, %** | **Missing-data handling** |
| KNHANES | Marital status | 32,935 | 32,712 | 223 | 0.68 | KNN imputation |
| KNHANES | PIR | 32,935 | 31,615 | 1,320 | 4.01 | KNN imputation |
| KNHANES | Education | 32,935 | 32,636 | 299 | 0.91 | KNN imputation |
| KNHANES | Smoking status | 32,935 | 32,398 | 537 | 1.63 | KNN imputation |
| KNHANES | Alcohol use | 32,935 | 32,174 | 761 | 2.31 | KNN imputation |
| KNHANES | Physical activity | 32,935 | 31,842 | 1,093 | 3.32 | KNN imputation |
| KNHANES | BMI | 32,935 | 32,390 | 545 | 1.65 | KNN imputation |
| KNHANES | Hypertension | 32,935 | 32,695 | 240 | 0.73 | KNN imputation |
| KNHANES | Diabetes mellitus | 32,935 | 32,618 | 317 | 0.96 | KNN imputation |
| KNHANES | Hyperlipidemia | 32,935 | 32,523 | 412 | 1.25 | KNN imputation |
| **Panel B. Patterns in NHANES** |  | |  |  |  |  |
| **Missingness pattern** | **Number of missing variables** | | **n** | | **%** | |
| Complete for all evaluated variables |  | 0 |  | 19,738 |  | 77.75 |
| Marital status only |  | 1 |  | 194 |  | 0.76 |
| PIR only |  | 1 |  | 622 |  | 2.45 |
| Education only |  | 1 |  | 200 |  | 0.79 |
| Smoking status only |  | 1 |  | 300 |  | 1.18 |
| Alcohol use only |  | 1 |  | 343 |  | 1.35 |
| Physical activity only |  | 1 |  | 497 |  | 1.96 |
| BMI only |  | 1 |  | 285 |  | 1.12 |
| Hypertension only |  | 1 |  | 182 |  | 0.72 |
|  |  |  |  |  |  |  |
| continued |  |  |  |  |  |  |
| **Missingness pattern** | **Number of missing variables** | | **n** | | **%** | |
| Diabetes mellitus only |  | 1 |  | 222 |  | 0.87 |
| Hyperlipidemia only |  | 1 |  | 248 |  | 0.98 |
| PIR + Physical activity |  | 2 |  | 633 |  | 2.49 |
| Alcohol use + Hyperlipidemia |  | 2 |  | 351 |  | 1.38 |
| Diabetes mellitus + Smoking status |  | 2 |  | 338 |  | 1.33 |
| BMI + PIR |  | 2 |  | 316 |  | 1.24 |
| Education + Hypertension |  | 2 |  | 187 |  | 0.74 |
| Alcohol use + Marital status |  | 2 |  | 172 |  | 0.68 |
| Marital status + Physical activity |  | 2 |  | 124 |  | 0.49 |
| BMI + Education |  | 2 |  | 118 |  | 0.46 |
| Hypertension + Smoking status |  | 2 |  | 91 |  | 0.36 |
| Hyperlipidemia + Smoking status |  | 2 |  | 27 |  | 0.11 |
| PIR + Physical activity + Alcohol use |  | 3 |  | 80 |  | 0.32 |
| PIR + Physical activity + BMI |  | 3 |  | 40 |  | 0.16 |
| PIR + Physical activity + Smoking status |  | 3 |  | 30 |  | 0.12 |
| PIR + Alcohol use + BMI |  | 3 |  | 20 |  | 0.08 |
| PIR + Physical activity + Hyperlipidemia |  | 3 |  | 20 |  | 0.08 |
| Physical activity + Alcohol use + Smoking status |  | 3 |  | 10 |  | 0.04 |
| **Panel C. Patterns in KNHANES** |  |  |  |  |  |  |
| **Missingness pattern** | **Number of missing variables** | | **n** | | **%** | |
| Complete for all evaluated variables |  | 0 |  | 30,244 |  | 91.83 |
| Marital status only |  | 1 |  | 19 |  | 0.06 |
| PIR only |  | 1 |  | 64 |  | 0.19 |
| Education only |  | 1 |  | 25 |  | 0.08 |
| Smoking status only |  | 1 |  | 37 |  | 0.11 |
| Alcohol use only |  | 1 |  | 36 |  | 0.11 |
| Physical activity only |  | 1 |  | 46 |  | 0.14 |
| continued |  |  |  |  |  |  |
| **Missingness pattern** | **Number of missing variables** | | **n** | | **%** | |
| BMI only |  | 1 |  | 36 |  | 0.11 |
| Hypertension only |  | 1 |  | 20 |  | 0.06 |
| Diabetes mellitus only |  | 1 |  | 24 |  | 0.07 |
| Hyperlipidemia only |  | 1 |  | 28 |  | 0.09 |
| PIR + Physical activity |  | 2 |  | 406 |  | 1.23 |
| Diabetes mellitus + Smoking status |  | 2 |  | 233 |  | 0.71 |
| BMI + PIR |  | 2 |  | 230 |  | 0.7 |
| Alcohol use + Hyperlipidemia |  | 2 |  | 222 |  | 0.67 |
| Alcohol use + Marital status |  | 2 |  | 133 |  | 0.4 |
| BMI + Education |  | 2 |  | 129 |  | 0.39 |
| Education + Hypertension |  | 2 |  | 115 |  | 0.35 |
| Hypertension + Smoking status |  | 2 |  | 85 |  | 0.26 |
| Hyperlipidemia + Smoking status |  | 2 |  | 52 |  | 0.16 |
| Marital status + Physical activity |  | 2 |  | 51 |  | 0.15 |
| PIR + Physical activity + Alcohol use |  | 3 |  | 220 |  | 0.67 |
| PIR + Physical activity + BMI |  | 3 |  | 100 |  | 0.3 |
| PIR + Physical activity + Smoking status |  | 3 |  | 90 |  | 0.27 |
| PIR + Alcohol use + Hyperlipidemia |  | 3 |  | 70 |  | 0.21 |
| PIR + Physical activity + Diabetes mellitus |  | 3 |  | 60 |  | 0.18 |
| Physical activity + Alcohol use + BMI |  | 3 |  | 50 |  | 0.15 |
| PIR + Smoking status + Hyperlipidemia |  | 3 |  | 40 |  | 0.12 |
| Physical activity + Education + Alcohol use |  | 3 |  | 30 |  | 0.09 |
| PIR + Physical activity + Hypertension |  | 3 |  | 20 |  | 0.06 |
| PIR + Physical activity + Marital status |  | 3 |  | 20 |  | 0.06 |

**Table S4** Sensitivity analyses of the indirect association through SII in the association between DII and CRAS.

| **Analysis** | **ACME (95% CI)** | **ADE (95% CI)** | **Total effect (95% CI)** | **Proportion mediated% (95% CI)** |
| --- | --- | --- | --- | --- |
| Model A | 6.48×10⁻⁵ (3.78×10⁻⁵, 1.04×10⁻⁴)*** | 9.67×10⁻⁴ (5.32×10⁻⁴, 1.34×10⁻³)*** | 1.03×10⁻³ (6.08×10⁻⁴, 1.41×10⁻³)*** | 6.08% (3.41%, 12.92%)*** |
| Model B | 4.63×10⁻⁵ (2.36×10⁻⁵, 7.91×10⁻⁵)*** | 8.14×10⁻⁴ (3.56×10⁻⁴, 1.22×10⁻³)*** | 8.61×10⁻⁴ (3.99×10⁻⁴, 1.26×10⁻³)*** | 5.19% (2.32%, 13.22%)*** |
| Model C | 4.41×10⁻⁵ (2.05×10⁻⁵, 7.33×10⁻⁵)*** | 7.83×10⁻⁴ (3.03×10⁻⁴, 1.19×10⁻³)** | 8.27×10⁻⁴ (3.49×10⁻⁴, 1.24×10⁻³)** | 5.26% (2.17%, 13.95%)** |
| Model C excluding extreme Ln_SII values | 3.41×10⁻⁵ (1.24×10⁻⁵, 6.18×10⁻⁵)*** | 6.76×10⁻⁴ (1.72×10⁻⁴, 1.13×10⁻³)** | 7.10×10⁻⁴ (2.12×10⁻⁴, 1.16×10⁻³)** | 4.63% (1.46%, 17.19%)** |

Note: Model A was adjusted for age, sex, ethnicity, marital status, poverty-income ratio, and education. Model B was additionally adjusted for smoking, alcohol consumption, physical activity, and BMI. Model C was further adjusted for hypertension, diabetes mellitus, and hyperlipidemia. In the extreme-value sensitivity analysis, participants with Ln_SII values below the 1st or above the 99th percentile were excluded. Adjusted GVIF values ranged from 1.03 to 1.41 in the mediator model and from 1.01 to 1.25 in the outcome model, indicating no substantial multicollinearity. ACME, average causal mediation effect; ADE, average direct effect; CI, confidence interval; SII, systemic immune-inflammation index.

**Table S5** Benjamini–Hochberg false discovery rate correction for exploratory analyses

| **Analysis family** | **Test** | **Nominal P value** | **FDR-adjusted P value** | **Significant after FDR correction** |
| --- | --- | --- | --- | --- |
| **DII subscore analyses, inflammatory-index associations, and indirect effects** |  |  |  |  |
| DII subscore analyses | NHANES DII_anti_abs | 0.051 | 0.051 | No |
| DII subscore analyses | NHANES DII_pro | 0.005 | 0.01 | Yes |
| DII subscore analyses | KNHANES DII_anti_abs | 0.008 | 0.011 | Yes |
| DII subscore analyses | KNHANES DII_pro | <0.001 | 0.003 | Yes |
| Inflammatory-index association | Ln_SIRI | <0.001 | 0.001 | Yes |
| Inflammatory-index association | Ln_NLR | <0.001 | 0.001 | Yes |
| Inflammatory-index association | Ln_SII | 0.021 | 0.021 | Yes |
| Inflammatory-index association | Ln_NAR | <0.001 | <0.001 | Yes |
| Inflammatory-index association | Ln_CALLY | <0.001 | 0.001 | Yes |
| Inflammatory-index association | Ln_IBI | <0.001 | 0.001 | Yes |
| Indirect effect (ACME) | NLR | <0.001 | <0.001 | Yes |
| Indirect effect (ACME) | SII | <0.001 | <0.001 | Yes |
| Indirect effect (ACME) | SIRI | <0.001 | <0.001 | Yes |
| Indirect effect (ACME) | NAR | <0.001 | <0.001 | Yes |
| Indirect effect (ACME) | CALLY | <0.001 | <0.001 | Yes |
| Indirect effect (ACME) | IBI | <0.001 | <0.001 | Yes |
|  |  |  |  |  |
| **Restricted cubic spline analyses** |  |  |  |  |
| Analysis family | Test | Nominal P value | FDR-adjusted P value | Significant after FDR correction |
| DII RCS overall | NHANES DII | <0.001 | <0.001 | Yes |
| DII RCS overall | KNHANES DII | <0.001 | <0.001 | Yes |
| DII RCS nonlinearity | NHANES DII | 0.331 | 0.331 | No |
| DII RCS nonlinearity | KNHANES DII | 0.011 | 0.022 | Yes |
| Inflammatory-index RCS overall | Ln_SIRI | <0.001 | <0.001 | Yes |
| Inflammatory-index RCS overall | Ln_NLR | <0.001 | <0.001 | Yes |
| Inflammatory-index RCS overall | Ln_SII | <0.001 | <0.001 | Yes |
| Inflammatory-index RCS overall | Ln_NAR | <0.001 | <0.001 | Yes |
| Inflammatory-index RCS overall | Ln_CALLY | <0.001 | <0.001 | Yes |
| Inflammatory-index RCS overall | Ln_IBI | <0.001 | <0.001 | Yes |
| Inflammatory-index RCS nonlinearity | Ln_SIRI | <0.001 | 0.001 | Yes |
| Inflammatory-index RCS nonlinearity | Ln_NLR | <0.001 | <0.001 | Yes |
| Inflammatory-index RCS nonlinearity | Ln_SII | <0.001 | <0.001 | Yes |
| Inflammatory-index RCS nonlinearity | Ln_NAR | <0.001 | 0.001 | Yes |
| Inflammatory-index RCS nonlinearity | Ln_CALLY | <0.001 | <0.001 | Yes |
| Inflammatory-index RCS nonlinearity | Ln_IBI | 0.006 | 0.006 | Yes |
|  |  |  |  |  |
|  |  |  |  |  |
| *Continued* |  |  |  |  |
| **Analysis family** | **Test** | **Nominal P value** | **FDR-adjusted P value** | **Significant after FDR correction** |
|  |  |  |  |  |
| **Subgroup interaction tests** |  |  |  |  |
| Cohort | Subgroup variable | P for interaction | FDR-adjusted P value | Significant after FDR correction |
| NHANES | Age | 0.08 | 0.452 | No |
| NHANES | Sex | 0.127 | 0.452 | No |
| NHANES | Ethnicity | 0.559 | 0.737 | No |
| NHANES | Marital status | 0.59 | 0.737 | No |
| NHANES | PIR | 0.58 | 0.737 | No |
| NHANES | Education | 0.041 | 0.452 | No |
| NHANES | Smoking status | 0.892 | 0.892 | No |
| NHANES | Drinking alcohol | 0.73 | 0.791 | No |
| NHANES | Physical activity | 0.624 | 0.737 | No |
| NHANES | BMI | 0.174 | 0.452 | No |
| NHANES | Hypertension | 0.384 | 0.713 | No |
| NHANES | Diabetes | 0.163 | 0.452 | No |
| NHANES | Hyperlipidemia | 0.359 | 0.713 | No |
| KNHANES | Age | 0.709 | 0.894 | No |
| KNHANES | Sex | 0.815 | 0.894 | No |
| KNHANES | Marital status | 0.621 | 0.894 | No |
| KNHANES | PIR | 0.662 | 0.894 | No |
| KNHANES | Education | 0.894 | 0.894 | No |
| KNHANES | Smoking status | 0.228 | 0.547 | No |
| KNHANES | Drinking alcohol | 0.205 | 0.547 | No |
| KNHANES | Physical activity | 0.117 | 0.547 | No |
| KNHANES | BMI | 0.175 | 0.547 | No |
| KNHANES | Hypertension | 0.019 | 0.228 | No |
| KNHANES | Diabetes | 0.861 | 0.894 | No |
| KNHANES | Hyperlipidemia | 0.307 | 0.614 | No |

**Table S6** Hyperparameter search spaces and final selected settings for the twelve machine-learning algorithms

| **Model** | **Hyperparameter search space** | **Final selected settings** |
| --- | --- | --- |
| Random Forest | n_estimators: 150–700; max_depth: None, 5, 10, or 20; min_samples_split: 2–20; min_samples_leaf: 1–10; max_features: sqrt, log2, or 0.7 | n_estimators=182; max_depth=5; min_samples_split=17; min_samples_leaf=4; max_features=log2 |
| XGBoost | n_estimators: 100–600; max_depth: 2–8; learning_rate: 0.01–0.20; subsample: 0.6–1.0; colsample_bytree: 0.6–1.0; min_child_weight: 1–8; reg_lambda: 0.1–10; scale_pos_weight: 1.000, 1.458, or 2.126 | n_estimators=210; max_depth=5; learning_rate=0.01731; subsample=0.7818; colsample_bytree=0.8041; min_child_weight=7; reg_lambda=8.5102; scale_pos_weight=1.458 |
| LightGBM | n_estimators: 100–800; max_depth: 2–8; learning_rate: 0.01–0.15; num_leaves: 8–64; min_child_samples: 20–150; subsample and colsample_bytree: 0.6–1.0; reg_alpha: 10⁻⁶–5; reg_lambda: 10⁻⁶–10 | n_estimators=680; max_depth=8; learning_rate=0.01; num_leaves=8; min_child_samples=36; subsample=0.6; colsample_bytree=0.6; reg_alpha=0.000460; reg_lambda=10 |
| Decision Tree | max_depth: 3–20; min_samples_split: 2–20; min_samples_leaf: 1–10; criterion: gini or entropy | max_depth=20; min_samples_split=3; min_samples_leaf=10; criterion=gini |
| KNN | n_neighbors: 3–15; weights: uniform or distance; p: 1 or 2 | n_neighbors=15; weights=distance; p=1 |
| GBDT | n_estimators: 80–400; learning_rate: 0.01–0.20; max_depth: 2–6; min_samples_split: 2–20; min_samples_leaf: 1–12; subsample: 0.6–1.0 | n_estimators=255; learning_rate=0.02036; max_depth=2; min_samples_split=20; min_samples_leaf=5; subsample=0.6011 |
| CatBoost | iterations: 100–600; depth: 3–9; learning_rate: 0.01–0.20; l2_leaf_reg: 0.5–12; random_strength: 0–1.5 | iterations=370; depth=4; learning_rate=0.01231; l2_leaf_reg=2.0351; random_strength=1.2748 |
| Logistic Regression | C: 0.005–20; penalty: L1 or L2 | C=0.26346; penalty=L2 |
| SVM | C: 0.01–20; kernel: linear or radial basis function; gamma: 10⁻⁴–1 | C=0.05529; kernel=linear; gamma=0.03244 |
| AdaBoost | n_estimators: 40–400; learning_rate: 0.01–1 | n_estimators=300; learning_rate=0.06923 |
| MLP | hidden-layer size: 40–160; alpha: 10⁻⁵–0.1; learning_rate_init: 0.0003–0.02; activation: ReLU or tanh | hidden-layer size=53; alpha=3.475×10⁻⁵; learning_rate_init=0.01793; activation=tanh |
| Gaussian Naive Bayes | var_smoothing: 10⁻¹¹–10⁻⁷ | var_smoothing=3.332×10⁻¹⁰ |

Note: The selected settings were obtained from the final five-fold Bayesian search fitted to the complete NHANES development training set. Integer bounds were inclusive. Learning rate and regularization ranges, as well as C, gamma, alpha, and var_smoothing, were sampled on a log-uniform scale. Each search used 20 iterations and optimized ROC-AUC. The XGBoost scale_pos_weight candidates corresponded to 1, the square root of the negative-to-positive class ratio, and the full class ratio in the NHANES development training set.

**Table S7** Baseline characteristics of participants stratified by Dietary Inflammatory Index quartiles in KNHANES

| Characteristic | Overall  N = 32935 | Q1  N = 8234 | Q2  N = 8234 | Q3  N =8233 | Q4  N = 8234 | p-value |
| --- | --- | --- | --- | --- | --- | --- |
| Age, years | 45.56± (16.46) | 47.72± (14.79) | 46.46± (15.81) | 45.42± (16.68) | 42.62± (17.99) | <0.001 |
| PIR | 2.73± (1.80) | 3.01± (1.90) | 2.80± (1.82) | 2.66± (1.75) | 2.45± (1.69) | <0.001 |
| Physical activity， (MET/week) | 1,562.41± (2,079.98) | 1,736.00± (2,222.26) | 1,640.33± (2,231.06) | 1,451.44± (1,850.08) | 1,419.90± (1,971.13) | <0.001 |
| BMI, kg/m2 | 23.91± (3.67) | 24.18± (3.46) | 23.89± (3.49) | 23.87± (3.74) | 23.69± (3.93) | <0.001 |
| DII | 0.37± (1.76) | -2.00± (0.80) | -0.17± (0.41) | 1.13± (0.36) | 2.52± (0.48) | <0.001 |
| Sex |  |  |  |  |  | <0.001 |
| Male | 13,993 (50.0%) | 4,644 (64.6%) | 3,828 (53.9%) | 3,150 (46.0%) | 2,371 (35.6%) |  |
| Female | 18,942 (50.0%) | 3,590 (35.4%) | 4,406 (46.1%) | 5,083 (54.0%) | 5,863 (64.4%) |  |
| Marital status |  |  |  |  |  | <0.001 |
| Married/  Living with Partner | 22,299 (63.1%) | 6,286 (72.2%) | 5,932 (67.5%) | 5,526 (62.2%) | 4,555 (50.5%) |  |
| Widowed/Divorced  /Separated | 4,111 (9.2%) | 756 (7.4%) | 884 (7.9%) | 1,085 (9.8%) | 1,386 (11.7%) |  |
| Never married | 6,525 (27.7%) | 1,192 (20.4%) | 1,418 (24.6%) | 1,622 (28.0%) | 2,293 (37.9%) |  |
| Education |  |  |  |  |  | <0.001 |
| Below high school | 8,584 (18.4%) | 1,630 (14.3%) | 2,029 (17.4%) | 2,374 (20.4%) | 2,551 (21.6%) |  |
| High school | 11,260 (37.0%) | 2,893 (35.9%) | 2,744 (35.8%) | 2,787 (37.3%) | 2,836 (39.1%) |  |
| Above high school | 13,091 (44.5%) | 3,711 (49.8%) | 3,461 (46.8%) | 3,072 (42.3%) | 2,847 (39.3%) |  |
| Smoking status |  |  |  |  |  | <0.001 |
| never | 21,467 (61.6%) | 4,682 (52.4%) | 5,225 (60.1%) | 5,589 (64.1%) | 5,971 (69.8%) |  |
| former | 6,447 (19.9%) | 2,231 (28.0%) | 1,732 (20.8%) | 1,405 (17.5%) | 1,079 (13.1%) |  |
| now | 5,021 (18.6%) | 1,321 (19.6%) | 1,277 (19.0%) | 1,239 (18.4%) | 1,184 (17.2%) |  |
|  |  |  |  |  |  |  |
|  |  |  |  |  |  |  |
|  |  |  |  |  |  |  |
|  |  |  |  |  |  |  |
| *Continued* |  |  |  |  |  |  |
| Characteristic | Overall  N = 32935 | Q1  N = 8234 | Q2  N = 8234 | Q3  N =8233 | Q4  N = 8234 | p-value |
| Drinking alcohol |  |  |  |  |  | 0.001 |
| never | 3,444 (7.9%) | 731 (6.8%) | 804 (7.5%) | 926 (8.3%) | 983 (8.9%) |  |
| former | 5,453 (14.5%) | 1,340 (14.5%) | 1,391 (15.1%) | 1,408 (14.9%) | 1,314 (13.4%) |  |
| mild | 19,317 (61.5%) | 4,939 (61.8%) | 4,886 (61.6%) | 4,769 (61.6%) | 4,723 (61.2%) |  |
| moderate | 3,369 (11.5%) | 866 (12.0%) | 822 (11.3%) | 816 (10.9%) | 865 (11.7%) |  |
| heavy | 1,352 (4.6%) | 358 (4.9%) | 331 (4.6%) | 314 (4.4%) | 349 (4.8%) |  |
| Hypertension |  |  |  |  |  | 0.004 |
| Yes | 7,453 (17.4%) | 1,821 (18.0%) | 1,815 (17.1%) | 1,977 (18.3%) | 1,840 (16.3%) |  |
| No | 25,482 (82.6%) | 6,413 (82.0%) | 6,419 (82.9%) | 6,256 (81.7%) | 6,394 (83.7%) |  |
| DM |  |  |  |  |  | <0.001 |
| No | 27,592 (86.4%) | 6,828 (85.0%) | 6,909 (86.8%) | 6,850 (85.9%) | 7,005 (87.8%) |  |
| Yes | 3,054 (7.2%) | 787 (7.7%) | 750 (7.0%) | 814 (7.6%) | 703 (6.4%) |  |
| Prediabetes | 2,289 (6.5%) | 619 (7.3%) | 575 (6.2%) | 569 (6.6%) | 526 (5.8%) |  |
| Hyperlipidemia |  |  |  |  |  | <0.001 |
| Yes | 6,180 (15.1%) | 1,673 (17.2%) | 1,612 (15.5%) | 1,538 (15.1%) | 1,357 (12.7%) |  |
| No | 26,755 (84.9%) | 6,561 (82.8%) | 6,622 (84.5%) | 6,695 (84.9%) | 6,877 (87.3%) |  |
| MI–CKD–anemia phenotype |  |  |  |  |  | <0.001 |
| Yes | 556 (1.2%) | 78 (0.7%) | 110 (0.9%) | 145 (1.2%) | 223 (1.8%) |  |
| No | 32,379 (98.8%) | 8,156 (99.3%) | 8,124 (99.1%) | 8,088 (98.8%) | 8,011 (98.2%) |  |

**Table S8** Baseline characteristic table based on CRAS grouping in NHANES

| **Characteristic** | **Overall**  (N = 25388) | **CRAS**  (N =239) | **Non-CRAS**  (N = 25149) | **p-value** |
| --- | --- | --- | --- | --- |
| DII | 0.89± (1.74) | 1.76± (1.52) | 0.89± (1.74) | <0.001 |
| Age, years | 46.17± (16.12) | 71.36± (11.00) | 46.04± (16.04) | <0.001 |
| PIR | 3.20± (1.61) | 2.20± (1.38) | 3.20± (1.61) | <0.001 |
| Physical activity， (MET/week) | 3,242.13± (5,402.82) | 754.25± (941.25) | 3,254.97± (5,413.36) | <0.001 |
| BMI, kg/m2 | 28.50± (6.41) | 31.36± (8.06) | 28.49± (6.40) | 0.001 |
| IBI | 7.51± (23.96) | 37.82± (72.61) | 7.36± (23.35) | <0.001 |
| CALLY | 103.13± (166.01) | 34.91± (103.02) | 103.49± (166.20) | <0.001 |
| SIRI | 1.23± (0.82) | 2.32± (1.71) | 1.23± (0.81) | <0.001 |
| NAR | 0.10± (0.04) | 0.13± (0.06) | 0.10± (0.04) | <0.001 |
| SII | 551.49± (320.65) | 862.31± (747.46) | 549.89± (316.19) | <0.001 |
| NLR | 2.17± (1.08) | 3.56± (2.28) | 2.17± (1.07) | <0.001 |
| Sex |  |  |  | 0.768 |
| Male | 13,350 (51.2%) | 138 (50.2%) | 13,212 (51.2%) |  |
| Female | 12,038 (48.8%) | 101 (49.8%) | 11,937 (48.8%) |  |
| Ethnicity |  |  |  | <0.001 |
| Non-Hispanic White | 12,832 (74.0%) | 118 (69.5%) | 12,714 (74.0%) |  |
| Non-Hispanic Black | 4,750 (9.0%) | 86 (22.4%) | 4,664 (8.9%) |  |
| Mexican American | 3,915 (6.7%) | 19 (3.2%) | 3,896 (6.7%) |  |
| Other Hispanic | 1,823 (4.7%) | 9 (2.4%) | 1,814 (4.7%) |  |
| Other Race | 2,068 (5.7%) | 7 (2.5%) | 2,061 (5.7%) |  |
| Marital status |  |  |  | <0.001 |
| Married/Living with Partner | 15,865 (66.3%) | 108 (46.5%) | 15,757 (66.4%) |  |
| Widowed/Divorced/Separated | 4,996 (16.5%) | 112 (46.5%) | 4,884 (16.3%) |  |
| Never married | 4,527 (17.3%) | 19 (7.0%) | 4,508 (17.3%) |  |
| Education |  |  |  | <0.001 |
| Below high school | 2,010 (3.6%) | 41 (12.4%) | 1,969 (3.6%) |  |
| High school | 8,905 (32.0%) | 112 (49.7%) | 8,793 (31.9%) |  |
| Above high school | 14,473 (64.4%) | 86 (37.9%) | 14,387 (64.6%) |  |
| Smoking status |  |  |  | <0.001 |
| never | 13,642 (54.2%) | 98 (41.9%) | 13,544 (54.3%) |  |
| former | 6,567 (25.6%) | 115 (47.8%) | 6,452 (25.5%) |  |
| now | 5,179 (20.2%) | 26 (10.4%) | 5,153 (20.3%) |  |
|  |  |  |  |  |
|  |  |  |  |  |
|  |  |  |  |  |
| *Continued* |  |  |  |  |
| **Characteristic** | **Overall**  (N = 25388) | **CRAS**  (N =239) | **Non-CRAS**  (N = 25149) | **p-value** |
| Drinking alcohol |  |  |  | <0.001 |
| never | 3,007 (9.6%) | 47 (18.8%) | 2,960 (9.6%) |  |
| former | 4,025 (13.0%) | 113 (49.7%) | 3,912 (12.8%) |  |
| mild | 9,089 (38.0%) | 63 (27.2%) | 9,026 (38.1%) |  |
| moderate | 4,142 (18.2%) | 10 (2.4%) | 4,132 (18.3%) |  |
| heavy | 5,125 (21.1%) | 6 (1.9%) | 5,119 (21.2%) |  |
| Hypertension |  |  |  | <0.001 |
| Yes | 10,043 (34.4%) | 216 (89.5%) | 9,827 (34.1%) |  |
| No | 15,345 (65.6%) | 23 (10.5%) | 15,322 (65.9%) |  |
| DM |  |  |  | <0.001 |
| No | 19,877 (82.8%) | 89 (42.1%) | 19,788 (83.0%) |  |
| Yes | 3,714 (10.7%) | 137 (51.8%) | 3,577 (10.4%) |  |
| Prediabetes | 1,797 (6.6%) | 13 (6.2%) | 1,784 (6.6%) |  |
| Hyperlipidemia |  |  |  | <0.001 |
| Yes | 18,081 (70.3%) | 204 (88.2%) | 17,877 (70.3%) |  |
| No | 7,307 (29.7%) | 35 (11.8%) | 7,272 (29.7%) |  |
| ALCOHOL | 11.03± (26.42) | 2.38± (12.15) | 11.07± (26.46) | <0.001 |
| VITB12 | 5.43± (6.46) | 4.50± (4.52) | 5.44± (6.47) | <0.001 |
| VITB6 | 2.12± (1.31) | 1.66± (0.88) | 2.12± (1.31) | <0.001 |
| BCAROTENE | 2,200.16± (4,007.74) | 1,702.21± (2,803.13) | 2,202.73± (4,012.88) | 0.023 |
| CAFFEINE | 183.87± (201.20) | 119.64± (130.13) | 184.20± (201.45) | <0.001 |
| CARB | 260.65± (113.75) | 202.63± (80.94) | 260.95± (113.82) | <0.001 |
| CHOLES | 294.50± (199.29) | 263.35± (193.18) | 294.67± (199.31) | 0.028 |
| KCAL | 2,181.66± (871.57) | 1,632.93± (604.74) | 2,184.49± (871.84) | <0.001 |
| TOTALFAT | 83.77± (40.28) | 63.77± (30.23) | 83.87± (40.30) | <0.001 |
| FIBER | 17.21± (9.38) | 13.66± (6.91) | 17.23± (9.39) | <0.001 |
| FOLICACID | 418.12± (229.62) | 335.46± (154.99) | 418.55± (229.86) | <0.001 |
| IRON | 15.67± (8.25) | 13.65± (6.74) | 15.68± (8.26) | 0.001 |
| MG | 307.75± (137.98) | 228.01± (99.55) | 308.16± (138.04) | <0.001 |
| MUFA | 30.31± (15.60) | 22.73± (11.29) | 30.34± (15.61) | <0.001 |
| NIACIN | 25.96± (13.11) | 19.48± (8.09) | 26.00± (13.12) | <0.001 |
| PROTEIN | 84.71± (36.78) | 63.57± (25.79) | 84.82± (36.80) | <0.001 |
| PUFA | 18.52± (10.23) | 14.40± (8.68) | 18.54± (10.24) | <0.001 |
| RIBOFLAVIN | 2.25± (1.15) | 1.78± (0.79) | 2.25± (1.15) | <0.001 |
| SATFAT | 27.40± (14.56) | 21.04± (11.40) | 27.43± (14.57) | <0.001 |
| SE | 115.46± (55.84) | 89.38± (39.27) | 115.60± (55.89) | <0.001 |
|  |  |  |  |  |
|  |  |  |  |  |
|  |  |  |  |  |
| *Continued* |  |  |  |  |
| **Characteristic** | **Overall**  (N = 25388) | **CRAS**  (N =239) | **Non-CRAS**  (N = 25149) | **p-value** |
| THIAMIN | 1.68± (0.85) | 1.38± (0.61) | 1.68± (0.85) | <0.001 |
| VITA | 690.00± (722.30) | 676.59± (811.92) | 690.07± (721.82) | 0.078 |
| VITC | 86.66± (82.78) | 81.87± (75.58) | 86.68± (82.82) | 0.427 |
| VITE | 6.58± (6.19) | 4.83± (4.37) | 6.59± (6.20) | <0.001 |
| ZN | 12.13± (7.51) | 9.69± (5.27) | 12.14± (7.52) | <0.001 |
| ALA | 1.67± (1.05) | 1.36± (0.90) | 1.68± (1.06) | <0.001 |
| EPA | 0.04± (0.12) | 0.04± (0.18) | 0.04± (0.12) | 0.002 |
| DHA | 0.08± (0.18) | 0.07± (0.22) | 0.08± (0.18) | 0.106 |
| LA | 16.36± (9.19) | 12.60± (7.78) | 16.37± (9.19) | <0.001 |
| AA | 0.15± (0.11) | 0.13± (0.09) | 0.15± (0.11) | 0.03 |

**Table S9** Baseline characteristics by MI–CKD–anemia phenotype in the KNHANES myocardial infarction subgroup.

| Characteristic | Overall N = 1304 | Phenotype  N = 556 | Non-Phenotype  N = 748 | p-value |
| --- | --- | --- | --- | --- |
| Age, years | 68.51± (10.35) | 72.15± (9.74) | 65.96± (10.01) | <0.001 |
| PIR | 1.97± (1.91) | 1.55± (1.52) | 2.26± (2.09) | <0.001 |
| Physical activity， (MET/week) | 1,098.79± (1,642.72) | 755.25± (808.90) | 1,339.80± (1,998.60) | <0.001 |
| BMI, kg/m2 | 24.46± (3.21) | 24.12± (3.43) | 24.69± (3.04) | 0.003 |
| DII | 0.57± (1.80) | 1.03± (1.72) | 0.24± (1.78) | <0.001 |
| sex |  |  |  | 0.086 |
| Male | 685 (57.3%) | 274 (53.9%) | 411 (59.7%) |  |
| Female | 619 (42.7%) | 282 (46.1%) | 337 (40.3%) |  |
| Marital status |  |  |  | <0.001 |
| Married/Living with Partner | 874 (69.6%) | 314 (59.2%) | 560 (77.0%) |  |
| Widowed/Divorced/  Separated | 398 (27.2%) | 231 (38.1%) | 167 (19.6%) |  |
| Never married | 32 (3.1%) | 11 (2.7%) | 21 (3.4%) |  |
| Education |  |  |  | <0.001 |
| Below high school | 820 (57.6%) | 400 (66.4%) | 420 (51.4%) |  |
| High school | 305 (25.9%) | 112 (23.7%) | 193 (27.5%) |  |
| Above high school | 179 (16.5%) | 44 (10.0%) | 135 (21.1%) |  |
| Smoking status |  |  |  | 0.306 |
| never | 712 (50.7%) | 319 (53.5%) | 393 (48.7%) |  |
| former | 422 (35.0%) | 172 (33.8%) | 250 (35.8%) |  |
| now | 170 (14.3%) | 65 (12.7%) | 105 (15.4%) |  |
| Drinking alcohol |  |  |  | <0.001 |
| never | 297 (20.3%) | 167 (26.9%) | 130 (15.7%) |  |
| former | 342 (25.6%) | 168 (30.3%) | 174 (22.3%) |  |
| mild | 539 (42.4%) | 186 (34.3%) | 353 (48.0%) |  |
| moderate | 76 (7.5%) | 16 (4.0%) | 60 (9.9%) |  |
| heavy | 50 (4.2%) | 19 (4.4%) | 31 (4.1%) |  |
| Hypertension |  |  |  | <0.001 |
| Yes | 822 (62.7%) | 411 (74.0%) | 411 (54.7%) |  |
| No | 482 (37.3%) | 145 (26.0%) | 337 (45.3%) |  |
| DM |  |  |  | <0.001 |
| No | 754 (57.7%) | 265 (47.8%) | 489 (64.6%) |  |
| Yes | 433 (33.5%) | 246 (43.4%) | 187 (26.5%) |  |
| Prediabetes | 117 (8.8%) | 45 (8.8%) | 72 (8.9%) |  |
|  |  |  |  |  |
|  |  |  |  |  |
| *Continued* |  |  |  |  |
| Characteristic | Overall N = 1304 | Phenotype  N = 556 | Non-Phenotype  N = 748 | p-value |
| Hyperlipidemia |  |  |  | 0.1 |
| Yes | 591 (45.0%) | 233 (41.9%) | 358 (47.1%) |  |
| No | 713 (55.0%) | 323 (58.1%) | 390 (52.9%) |  |
| BCAROTENE | 3,020.90± (3,585.38) | 2,331.05± (2,504.28) | 3,504.87± (4,113.31) | <0.001 |
| CARB | 273.39± (109.86) | 250.28± (98.94) | 289.61± (114.22) | <0.001 |
| CHOLES | 172.31± (182.96) | 140.21± (166.35) | 194.83± (190.68) | <0.001 |
| KCAL | 1,706.16± (754.08) | 1,520.83± (612.55) | 1,836.17± (814.83) | <0.001 |
| TOTALFAT | 33.97± (27.87) | 28.91± (22.80) | 37.51± (30.45) | <0.001 |
| FIBER | 25.79± (13.67) | 22.95± (12.64) | 27.78± (14.01) | <0.001 |
| FOLICACID | 316.87± (163.06) | 278.23± (149.03) | 343.98± (167.05) | <0.001 |
| IRON | 10.73± (7.27) | 8.57± (5.73) | 12.24± (7.83) | <0.001 |
| MG | 307.94± (144.47) | 268.75± (129.02) | 335.43± (148.41) | <0.001 |
| MUFA | 10.63± (10.49) | 8.90± (8.40) | 11.84± (11.59) | <0.001 |
| NIACIN | 11.27± (6.87) | 9.38± (5.37) | 12.60± (7.48) | <0.001 |
| PROTEIN | 59.84± (32.84) | 52.12± (27.39) | 65.27± (35.19) | <0.001 |
| PUFA | 9.36± (8.05) | 8.33± (7.24) | 10.08± (8.51) | <0.001 |
| RIBOFLAVIN | 1.29± (0.83) | 1.09± (0.67) | 1.43± (0.90) | <0.001 |
| SATFAT | 10.14± (8.93) | 8.50± (6.89) | 11.29± (9.96) | <0.001 |
| THIAMIN | 1.19± (0.78) | 0.98± (0.60) | 1.35± (0.85) | <0.001 |
| VITA | 495.92± (745.30) | 346.03± (449.40) | 601.07± (881.62) | <0.001 |
| VITC | 65.36± (72.77) | 55.60± (72.75) | 72.21± (72.04) | <0.001 |
| VITE | 5.77± (3.52) | 5.21± (3.82) | 6.17± (3.24) | <0.001 |
| ZN | 9.49± (4.74) | 8.36± (3.80) | 10.28± (5.16) | <0.001 |
| N3 | 1.75± (2.40) | 1.55± (2.09) | 1.89± (2.59) | <0.001 |
| N6 | 7.60± (6.55) | 6.75± (5.97) | 8.20± (6.87) | <0.001 |

**Table S10** The associations between Six Systemic Inflammatory Indices and CRAS in weighted logistic regression models.

|  | Model1 | | Model2 | | Model3 | |
| --- | --- | --- | --- | --- | --- | --- |
|  | OR(95% CI) | P | OR(95% CI) | P | OR(95% CI) | P |
| Ln_SIRI | 6.01 (4.88, 7.40) | <0.001 | 4.46 (3.65, 5.46) | <0.001 | 4.24 (3.34, 5.39) | <0.001 |
| Q1(ref) | 1 |  | 1 |  | 1 |  |
| Q2 | 1.85 (0.84, 4.06) | 0.126 | 2.23 (1.01, 4.91) | 0.047 | 2.05 (0.92, 4.56) | 0.080 |
| Q3 | 3.10 (1.50, 6.38) | 0.002 | 3.38 (1.62, 7.08) | 0.001 | 2.72 (1.27, 5.82) | 0.010 |
| Q4 | 24.46 (13.77, 43.44) | <0.001 | 20.36 (10.75, 38.58) | <0.001 | 15.92 (8.15, 31.09) | <0.001 |
| P for trend | <0.001 |  | <0.001 |  | <0.001 |  |
|  |  |  |  |  |  |  |
| Ln_NLR | 6.55 (4.68, 9.16) | <0.001 | 3.78 (2.57, 5.57) | <0.001 | 3.44 (2.27, 5.23) | <0.001 |
| Q1(ref) | 1 |  | 1 |  | 1 |  |
| Q2 | 1.40 (0.77, 2.57) | 0.264 | 1.69 (0.90, 3.17) | 0.101 | 1.47 (0.76, 2.83) | 0.245 |
| Q3 | 1.89 (1.05, 3.40) | 0.032 | 2.19 (1.20, 4.00) | 0.01 | 1.75 (0.94, 3.25) | 0.075 |
| Q4 | 7.64 (4.66, 12.55) | <0.001 | 6.05 (3.40, 10.74) | <0.001 | 4.80 (2.62, 8.80) | <0.001 |
| P for trend | <0.001 |  | <0.001 |  | <0.001 |  |
|  |  |  |  |  |  |  |
| Ln_SII | 2.13 (1.31, 3.48) | 0.002 | 1.75 (1.13, 2.70) | 0.011 | 1.71 (1.08, 2.69) | 0.021 |
| Q1(ref) | 1 |  | 1 |  | 1 |  |
| Q2 | 0.482 (0.239, 0.975) | 0.042 | 0.583 (0.285, 1.192) | 0.138 | 0.551 (0.266, 1.141) | 0.107 |
| Q3 | 0.919 (0.508, 1.662) | 0.778 | 1.131 (0.592, 2.160) | 0.706 | 1.040 (0.522, 2.071) | 0.911 |
| Q4 | 2.474 (1.624, 3.768) | <0.001 | 2.387 (1.482, 3.846) | <0.001 | 2.224 (1.343, 3.683) | 0.002 |
| P for trend | <0.001 |  | <0.001 |  | <0.001 |  |
|  |  |  |  |  |  |  |
| Ln_NAR | 5.54 (3.75, 8.16) | <0.001 | 5.93 (3.87, 9.10) | <0.001 | 3.88 (2.38, 6.33) | <0.001 |
| Q1(ref) | 1 |  | 1 |  | 1 |  |
| Q2 | 1.35 (0.76, 2.38) | 0.300 | 1.38 (0.77, 2.49) | 0.273 | 1.10 (0.59, 2.01) | 0.761 |
| Q3 | 2.62 (1.45, 4.73) | 0.001 | 2.48 (1.37, 4.49) | 0.002 | 1.81 (0.98, 3.34) | 0.055 |
| Q4 | 4.53 (2.77, 7.42) | <0.001 | 4.50 (2.68, 7.55) | <0.001 | 2.64 (1.50, 4.64) | 0.001 |
| P for trend | <0.001 |  | <0.001 |  | < 0.001 |  |
|  |  |  |  |  |  |  |
| Ln_CALLY | 0.36 (0.32, 0.41) | <0.001 | 0.41 (0.35, 0.48) | <0.001 | 0.46 (0.39, 0.55) | <0.001 |
| Q1(ref) | 1 |  | 1 |  | 1 |  |
| Q2 | 0.20 (0.13, 0.30) | <0.001 | 0.25 (0.16, 0.39) | <0.001 | 0.32 (0.20, 0.50) | <0.001 |
| Q3 | 0.07 (0.04, 0.13) | <0.001 | 0.12 (0.07, 0.22) | <0.001 | 0.19 (0.10, 0.35) | <0.001 |
| Q4 | 0.06 (0.03, 0.12) | <0.001 | 0.18 (0.09, 0.36) | <0.001 | 0.30 (0.15, 0.60) | 0.001 |
| P for trend | <0.001 |  | <0.001 |  | <0.001 |  |
|  | | | | | | |
|  |  |  |  |  |  |  |
| *Continued* |  | | | | | |
|  | Model1 | | Model2 | | Model3 | |
| Ln_IBI | 2.35 (2.12, 2.61) | <0.001 | 2.20 (1.92, 2.52) | <0.001 | 1.94 (1.65, 2.27) | <0.001 |
| Q1(ref) | 1 |  | 1 |  | 1 |  |
| Q2 | 1.47 (0.62, 3.45) | 0.379 | 0.90 (0.38, 2.15) | 0.813 | 0.80 (0.33, 1.94) | 0.619 |
| Q3 | 3.08 (1.47, 6.46) | 0.003 | 1.52 (0.72, 3.22) | 0.27 | 1.11 (0.53, 2.34) | 0.777 |
| Q4 | 13.34 (6.85, 25.96) | <0.001 | 5.61 (2.86, 11.02) | <0.001 | 3.08 (1.54, 6.19) | 0.002 |
| P for trend | <0.001 |  | <0.001 |  | <0.001 |  |

Three hierarchical models were constructed with sequential covariate adjustment:

Model I: Unadjusted;

Model II: Adjusted for demographic variables;

Model III: Adjusted for all covariates.

**Table S11** Sensitivity analysis of the association between DII and CRAS

| Methods |  | Model1 | | Model2 | | Model3 | |
| --- | --- | --- | --- | --- | --- | --- | --- |
|  |  | OR(95% CI) | P | OR(95% CI) | P | OR(95% CI) | P |
| Multiple imputation | DII | 1.40(1.26,1.55) | <0.001 | 1.38(1.23,1.54) | <0.001 | 1.30(1.16,1.46) | 0.004 |
|  | Q1(ref) | 1.00 |  | 1.00 |  | 1.00 |  |
|  | Q2 | 1.45(0.71,2.94) | 0.310 | 1.43(0.71,2.89) | 0.322 | 1.33(0.66,2.69) | 0.426 |
|  | Q3 | 4.63(2.66,8.07) | <0.001 | 4.46(2.53,7.88) | <0.001 | 4.00(2.22,7.20) | <0.001 |
|  | Q4 | 4.95(2.85,8.60) | <0.001 | 4.50(2.55,7.96) | <0.001 | 3.64(2.07,6.41) | <0.001 |
|  | DII_anti | 0.48(0.31,0.73) | <0.001 | 0.51(0.33,0.78) | 0.002 | 0.57(0.37,0.86) | 0.008 |
|  | DII_pro | 1.49(1.32,1.68) | <0.001 | 1.47(1.28,1.68) | <0.001 | 1.37(1.19,1.58) | <0.001 |
|  | P for trend | <0.001 |  | <0.001 |  | <0.001 |  |
|  |  |  | |  | |  | |
| Exclude extreme BMI | DII | 1.37(1.23,1.53) | <0.001 | 1.29(1.13,1.47) | <0.001 | 1.30(1.06,1.38) | 0.006 |
|  | Q1(ref) | 1.00 |  | 1.00 |  | 1.00 |  |
|  | Q2 | 1.38(0.69,2.75) | 0.359 | 1.29(0.66,2.54) | 0.459 | 1.13(0.55,2.29) | 0.741 |
|  | Q3 | 3.27(2.03,5.27) | <0.001 | 2.92(1.78,4.79) | <0.001 | 2.39(1.46,3.94) | <0.001 |
|  | Q4 | 4.03(2.48,6.55) | <0.001 | 3.07(1.84,5.10) | <0.001 | 2.27(1.35,3.81) | 0.002 |
|  | DII_anti | 0.54(0.36,0.83) | <0.001 | 0.62(0.41,0.92) | 0.017 | 0.72(0.49,1.06) | 0.09 |
|  | DII_pro | 1.47(1.29,1.67) | <0.001 | 1.37(1.17,1.61) | <0.001 | 1.26(1.07,1.50) | 0.008 |
|  | P for trend | <0.001 |  | <0.001 |  | 0.002 |  |
|  |  |  | |  | |  | |
| More covariates | DII | 1.38(1.24,1.54) | <0.001 | 1.18(1.02,1.36) | 0.022 | 1.18(1.03,1.36) | 0.019 |
|  | Q1(ref) | 1.00 |  | 1.00 |  | 1.00 |  |
|  | Q2 | 1.44(0.73,2.81) | 0.290 | 1.12(0.56,2.26) | 0.742 | 1.12(0.55,2.26) | 0.76 |
|  | Q3 | 3.38(2.10,5.43) | <0.001 | 1.99(1.15,3.44) | 0.014 | 2.03(1.16,3.55) | 0.014 |
|  | Q4 | 4.31(2.67,6.96) | <0.001 | 2.14(1.26,3.63) | 0.005 | 2.17(1.27,3.71) | 0.005 |
|  | DII_anti_abs | 0.53(0.35,0.81) | <0.001 | 0.79(0.53,1.17) | 0.237 | 0.78(0.52,1.18) | 0.242 |
|  | DII_pro | 1.48(1.31,1.68) | <0.001 | 1.23(1.03,1.48) | 0.022 | 1.24(1.04,1.48) | 0.017 |
|  | P for trend | <0.001 |  | 0.006 |  | 0.004 |  |

**Multiple interpolation**: Missing values were handled using multiple imputation.

**Exclude extreme BMI**: participants with extreme BMI values were excluded;

**More covariates**: complete blood count and blood biochemistry parameters were additionally included as covariates in the fully adjusted model: Albumin (g/dL), C-reactive protein , CRP (mg/dL), Lymphocyte (10⁹/L), Monocyte(10⁹/L), Neutrophils(10⁹/L) and Platelet(×10⁹/L).

**Table S12** Sensitivity analysis of the association between the shared 22-component Dietary Inflammatory Index and CRAS in NHANES.

|  | Model1 | | Model2 | | Model3 | |
| --- | --- | --- | --- | --- | --- | --- |
|  | OR (95% CI) | P | OR (95% CI) | P | OR (95% CI) | P |
| NHANES |  |  |  |  |  |  |
| DII_22 | 1.34(1.20,1.50) | <0.001 | 1.28(1.13,1.45) | <0.001 | 1.21(1.06,1.39) | 0.005 |
| Q1(ref) | 1 |  | 1 |  | 1 |  |
| Q2 | 1.90(1.10,3.29) | 0.020 | 1.77(1.02,3.07) | 0.041 | 1.57(0.88,2.81) | 0.122 |
| Q3 | 3.51(2.04,6.04) | <0.001 | 3.15(1.80,5.49) | <0.001 | 2.69(1.56,4.64) | <0.001 |
| Q4 | 3.83(2.19,6.71) | <0.001 | 3.09(1.72,5.57) | <0.001 | 2.50(1.36,4.59) | 0.003 |
| DII_anti_abs | 0.54(0.37,0.78) | 0.001 | 0.59(0.42,0.85) | 0.004 | 0.67(0.47,0.95) | 0.024 |
| DII_pro | 1.45(1.26,1.67) | <0.001 | 1.36(1.14,1.62) | <0.001 | 1.27(1.04,1.54) | 0.017 |
| P for trend |  | <0.001 |  | <0.001 |  | 0.004 |

DII_22 was recalculated in NHANES using the 22 dietary parameters available in both NHANES and KNHANES. The corresponding KNHANES estimates remain those presented in Table 2.

**Figure S2** Restricted cubic spline analysis of the association between the shared 22-component DII and CRAS in NHANES.

The DII was recalculated using the 22 dietary parameters shared by NHANES and KNHANES. The model was adjusted for all covariates included in Model III. The solid line represents the odds ratio, and the shaded area represents the 95% confidence interval.

A	B

**Figure S3** Precision–recall curves for the 12 machine-learning models. (A) Prediction of HF-defined CRAS in the NHANES internal validation set. (B) Exploratory cross-phenotype application to the MI–CKD–anemia phenotype in KNHANES. The horizontal dashed line in each panel represents the observed outcome prevalence in the corresponding evaluation sample. AP denotes average precision.

**Table S13** Performance of CatBoost and clinically defined benchmark models in the NHANES internal validation set and KNHANES cross-phenotype application.

| **Model** | **Accuracy** | **Sensitivity** | **Specificity** | **F1-score** | **ROC-AUC (95% CI)** | **PR-AUC** | **Brier score** |
| --- | --- | --- | --- | --- | --- | --- | --- |
| **NHANES** |  |  |  |  |  |  |  |
| CatBoost | 0.656 | 0.625 | 0.671 | 0.539 | 0.741 (0.673–0.805) | 0.556 | 0.186 |
| Age-only logistic regression | 0.607 | 0.514 | 0.651 | 0.457 | 0.637 (0.560–0.714) | 0.433 | 0.210 |
| Conventional clinical logistic regression | 0.652 | 0.625 | 0.665 | 0.536 | 0.679 (0.605–0.750) | 0.479 | 0.202 |
| **KNHANES** |  |  |  |  |  |  |  |
| CatBoost | 0.668 | 0.745 | 0.611 | 0.657 | 0.753 (0.726–0.777) | 0.698 | 0.200 |
| Age-only logistic regression | 0.673 | 0.626 | 0.709 | 0.620 | 0.717 (0.690–0.745) | 0.659 | 0.226 |
| Conventional clinical logistic regression | 0.653 | 0.707 | 0.612 | 0.634 | 0.729 (0.70–0.757) | 0.675 | 0.212 |

Note: The age-only model included age as its sole predictor. The conventional clinical logistic regression model included age, BMI, sex, smoking status, hypertension, diabetes mellitus, and hyperlipidemia. Both benchmark models were fitted using ordinary unpenalized logistic regression without LASSO or resampling. Model-specific thresholds were determined using Youden’s J statistic from five-fold out-of-fold predictions in the NHANES development training set and were applied unchanged to both evaluation settings. ROC-AUC confidence intervals were estimated using 2,000 participant-level bootstrap resamples. PR-AUC was calculated using average precision. Lower Brier scores indicate better probabilistic accuracy. No model recalibration was performed in KNHANES.

**Table S14** Paired differences in predictive performance between CatBoost and the clinically defined benchmark models.

| **Dataset** | **Comparator** | **ΔROC-AUC (95% CI)** | **ΔPR-AUC (95% CI)** | **Brier score reduction (95% CI)** |
| --- | --- | --- | --- | --- |
| NHANES internal validation set | Age-only logistic regression | 0.104 (0.027–0.181) | 0.124 (0.020–0.232) | 0.024 (0.004–0.045) |
|  | Conventional clinical logistic regression | 0.062 (-0.015–0.138) | 0.077 (-0.028–0.188) | 0.016 (-0.004–0.036) |
| KNHANES cross-phenotype application | Age-only logistic regression | 0.035 (0.011–0.058) | 0.038 (0.003–0.073) | 0.026 (0.017–0.034) |
|  | Conventional clinical logistic regression | 0.024 (0.0001–0.048) | 0.023 (-0.015–0.059) | 0.012 (0.003–0.020) |

Note: Differences and percentile 95% confidence intervals were estimated using 2,000 paired participant-level bootstrap resamples. ΔROC-AUC and ΔPR-AUC were calculated as CatBoost minus the comparator. Brier score reduction was calculated as the comparator minus CatBoost. Positive values therefore indicate better performance by CatBoost.

Figure S4. Receiver operating characteristic and precision–recall curves comparing CatBoost with the age-only and conventional clinical logistic regression models. (A) ROC curves in the NHANES internal validation set; (B) precision–recall curves in the NHANES internal validation set; (C) ROC curves in the KNHANES cross-phenotype application; and (D) precision–recall curves in the KNHANES cross-phenotype application.

**Table S15** Bootstrap stability of global SHAP feature-importance rankings for the CatBoost model in the NHANES internal validation set.

| **Feature** | **Mean absolute SHAP (95% CI)** | **Point-estimate rank** | **Median bootstrap rank** | **Top-three frequency, %** |
| --- | --- | --- | --- | --- |
| Physical activity | 0.4889 (0.4517–0.5253) | 1 | 1 | 100 |
| Age | 0.4279 (0.3962–0.4570) | 2 | 2 | 100 |
| Drinking alcohol | 0.3851 (0.3678–0.4029) | 3 | 3 | 100 |
| Riboflavin intake | 0.1621 (0.1497–0.1731) | 4 | 4 | 0 |
| Marital status | 0.1599 (0.1424–0.1779) | 5 | 5 | 0 |
| Magnesium intake | 0.1407 (0.1286–0.1527) | 6 | 6 | 0 |
| Hypertension | 0.1276 (0.1106–0.1477) | 7 | 7 | 0 |
| Poverty-income ratio | 0.0878 (0.0692–0.1075) | 8 | 8 | 0 |

Note: Global feature importance was quantified as the mean absolute SHAP value among 224 participants in the NHANES internal validation set. Percentile 95% confidence intervals, median ranks, and top-three frequencies were estimated using 1,000 participant-level bootstrap resamples. The analysis was conditional on the fixed fitted CatBoost model and did not repeat feature selection or model fitting. Higher mean absolute SHAP values indicate greater average contribution magnitude
